# Proportion of Women and Reporting of Outcomes by Sex in Clinical Trials for Alzheimer Disease: A Systematic Review and Meta-analysis

**DOI:** 10.1001/jamanetworkopen.2021.24124

**Published:** 2021-09-13

**Authors:** Julie Martinkova, Frances-Catherine Quevenco, Helene Karcher, Alberto Ferrari, Else Charlotte Sandset, Cassandra Szoeke, Jakub Hort, Reinhold Schmidt, Antonella Santuccione Chadha, Maria Teresa Ferretti

**Affiliations:** 1 Memory Clinic, Department of Neurology, Second Faculty of Medicine, Charles University and Motol University Hospital, Prague, Czech Republic; 2 Women’s Brain Project, Guntershausen, Switzerland; 3 Roche Diagnostics International Ltd, Rotkreuz, Switzerland; 4 Novartis Pharma AG, Basel, Switzerland; 5 Stroke Unit, Department of Neurology, University of Oslo, Oslo, Norway; 6 Faculty of Medicine, Dentistry and Health Sciences, University of Melbourne, Melbourne, Australia; 7 International Clinical Research Center, St Anne’s University Hospital Brno, Brno, Czech Republic; 8 Department of Neurogeriatrics, University Clinic of Neurology, Medical University Graz, Graz, Austria; 9 Biogen International GMBH, Baar, Switzerland

## Abstract

**Importance:**

Women represent two-thirds of patients with Alzheimer disease (AD), and sex differences might affect results of randomized clinical trials (RCTs). However, little information exists on differences in sex as reported in RCTs for AD.

**Objective:**

To assess the ratio of females to males and the reporting of sex-stratified data in large pharmaceutical RCTs for AD.

**Data Sources:**

A search for pharmaceutical RCTs for AD was conducted on September 4, 2019, using ClinicalTrials.gov with the key word *Alzheimer disease*, and articles related to those trials were identified using the PubMed, Scopus, and Google Scholar databases. Searches were conducted between September 4 and October 31, 2019, and between April 15 and May 31, 2020.

**Study Selection:**

Controlled RCTs that had more than 100 participants and tested the efficacy of drugs or herbal extracts were included. Of 1047 RCTs identified, 409 were published and therefore screened. A total of 77 articles were included in the final analysis, including 56 primary articles on AD, 13 secondary articles on AD, and 8 articles on mild cognitive impairment.

**Data Extraction and Synthesis:**

The location and date of publication; number, sex, and age of patients enrolled; disease severity; experimental or approved status of the drug; and whether the study included a sex-stratified analysis in the protocol, methods, or results were extracted by 1 reviewer for each article, and the meta-analysis followed the Preferred Reporting Items for Systematic Reviews and Meta-analyses (PRISMA) reporting guideline. Data were analyzed using a mixed-effects model.

**Main Outcomes and Measures:**

The mean proportion of women enrolled in the trials and the associations between prespecified variables were analyzed. The proportion of articles that included sex-stratified results and the temporal trends in the reporting of these results were also studied.

**Results:**

In this review of 56 RCTs for AD involving 39 575 participants, 23 348 women (59.0%) were included. The mean (SD) proportion of women in RCTs of approved drugs was 67.3% (6.9%), and in RCTs of experimental drugs was 57.9% (5.9%). The proportion of women in RCTs of experimental drugs was significantly lower than the proportion of women in the general population with AD in the US (62.1%; difference, −4.56% [95% CI, −6.29% to −2.87%]; *P* < .001) and Europe (68.2%; difference, −10.67% [95% CI, −12.39% to −8.97%]; *P* < .001). Trials of approved drugs had a higher probability of including women than trials of experimental drugs (odds ratio [OR], 1.26; 95% CI, 1.05-1.52; *P* = .02). Both the severity of AD at baseline and the trial location were associated with the probability of women being enrolled in trials (severity: OR, 0.98; 95% CI, 0.97-1.00; *P* = .02; location in Europe: OR, 1.26; 95% CI, 1.05-1.52; *P* = .01; location in North America: OR, 0.81; 95% CI, 0.71-0.93; *P* = .002). Only 7 articles (12.5%) reported sex-stratified results, with an increasing temporal trend (*R*, 0.30; 95% CI, 0.05-0.59; *P* = .03).

**Conclusions and Relevance:**

In this systematic review and meta-analysis, the proportion of women in RCTs for AD, although higher than the proportion of men, was significantly lower than that in the general population. Only a small proportion of trials reported sex-stratified results. These findings support strategies to improve diversity in enrollment and data reporting in RCTs for AD.

## Key Points

**Question:**

What is the proportion of women in large randomized clinical trials for Alzheimer disease (AD), and are sex differences reported?

**Findings:**

In this systematic review and meta-analysis of 56 randomized clinical trials for AD with 39 575 total participants, 59.0% of patients overall and a mean of 57.9% in trials of experimental drugs were women, significantly lower proportions of women than in the US and European population with AD. Only 12.5% of articles reported sex-stratified results, but this proportion appeared to increase over time.

**Meaning:**

Although the findings suggest that current clinical trials for AD enroll more women than men, strategies to increase women’s participation in clinical trials for AD should be discussed and the reporting of trial outcomes by sex should be encouraged.

## Teaser

This systematic review and meta-analysis examines the ratio of males to females and the prevalence of sex-stratified data in published reports of large pharmaceutical randomized clinical trials for Alzheimer disease.

## Introduction

Alzheimer disease (AD) is the leading cause of dementia in the older population and affects more than 50 million individuals worldwide.^1^ Current treatment of AD is symptomatic and at the time of this study was based on 4 approved pharmaceuticals (galantamine, rivastigmine, donepezil, and memantine).

Substantial heterogeneity in risk factors, presentation, and progression among patients has hindered the clinical development of precise diagnostics and disease-modifying therapies.^2,3^ Sex differences are potential causes of disease heterogeneity. Women represent most patients with AD and related dementias (a mean of 68.2% of patients with AD in Europe and 62.1% in the US^4,5^). In addition, sex-related differences occur in disease symptoms, progression, and biomarkers^6,7,8,9,10,11,12,13,14^ and in genetic risk associated with the apolipoprotein E ɛ4 (*APOE4*) allele.^15,16^

These differences between men and women are likely associated with the efficacy of a tested drug in randomized clinical trials (RCTs). However, in RCTs among patients with AD, little attention has been given to the role of sex and gender differences. In a meta-analysis of 48 trials of approved AD therapeutics, trials enrolled more women than men (a mean of 63.8% women per trial), but none of the trials reported sex-stratified data.^17^

In contrast to RCTs for approved drugs, an overview of several phase 3 RCTs for experimental AD drugs reported that some trials enrolled approximately 50% men and 50% women.^7^ Whether and how sex is considered in current trials of experimental drugs remain to be established systematically. Therefore, we performed a systematic review and meta-analysis of articles related to RCTs for AD to examine the sex distribution of patients in the RCTs, the proportion of articles that reported sex-stratified data, and temporal trends in the findings.

## Methods

### Identification of Trials and Definition of Primary vs Secondary Articles

**Figure 1.**
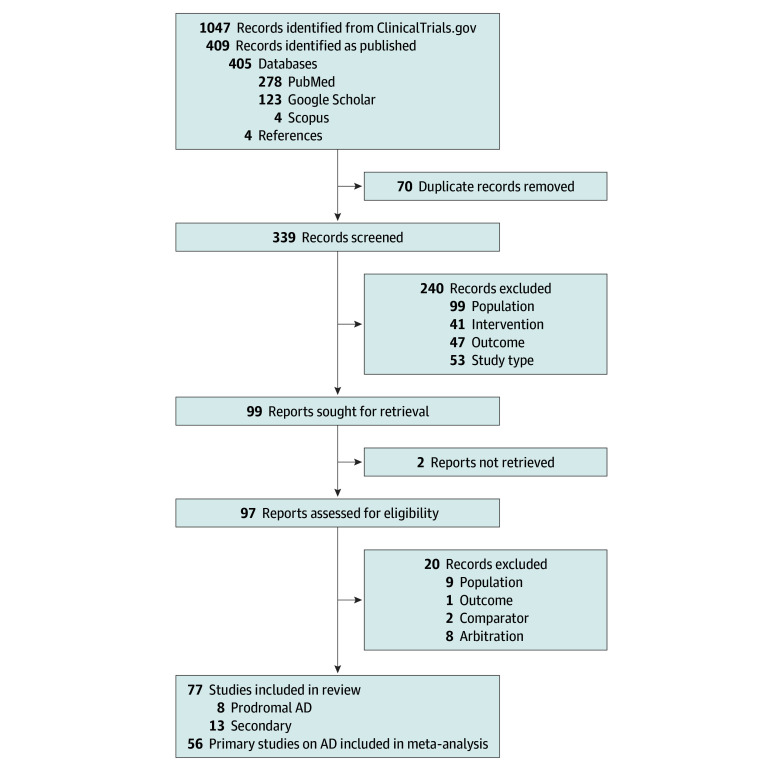
Article Selection Flowchart Primary articles were defined as the chronologically first article containing the main results of a trial; all other articles on the trial were considered secondary. AD indicates Alzheimer disease.

This systematic review and meta-analysis was conducted according to a prespecified protocol (reviewregistry855). We used a systematic stepwise approach similar to that used in previous articles (Figure 1).^18,19,20^ This study followed the Preferred Reporting Items for Systematic Reviews and Meta-analyses (PRISMA) reporting guideline.

First, we identified RCTs using the keyword *Alzheimer disease* at ClinicalTrials.gov, a large, web-based database resource maintained by the National Institutes of Health. All clinical trials performed in the US or to be used for US Food and Drug Administration submissions must be registered on ClinicalTrials.gov, but the database also includes trials not conducted in the US, making it one of the most complete trial databases. Only interventional trials in phase 1, 2, or 3 were included. The search was conducted on September 4, 2019.

Next, articles related to the RCTs found at ClinicalTrials.gov were identified by searching publicly available databases. PubMed was searched by one of us (F.C.Q.) on September 4, 2019, using the national clinical trial identifier, the principal investigator, and/or the trial name. Google Scholar and Scopus were searched by one of us (J.M.) between September 4 and October 31, 2019, and between April 15 and May 31, 2020, using each trial’s national clinical trial identifier and, when available, principal investigator and trial name. In addition, the reference list of each identified article was searched for other relevant trials. Further details on the search strategy are provided in eAppendix 1 the Supplement.

In cases in which several peer-reviewed articles had been published on the same trial, the chronologically first publication of the main results was considered as the primary article; the others, considered secondary articles, were subject to the same selection and extraction process but were not included in the meta-analysis.

### Selection of Trials

Published trials were selected according to predefined inclusion and exclusion criteria (eTable 1 in the Supplement) by 2 independent reviewers (J.M., M.T.F.). In case of disagreement, a third reviewer (A.S.C.) adjudicated. Only RCTs with more than 100 participants that included both sexes and enrolled patients with AD dementia or biomarker-confirmed mild cognitive impairment owing to AD (also referred to as *prodromal AD*) were selected. We included only RCTs studying the clinical efficacy of pharmacological, biological, or genetic agents or herbal extracts. Because efficacy is assessed even in early stages of trials, we did not limit our selection by trial phase. We focused on trials with more than 100 individuals because they are the most informative for the assessment of the benefit and safety profile of a given compound and they inform clinical practice; they also allow the robust calculation of the effects of sex. Because the scope of this review was to inform pharmaceutical RCT design, we excluded behavioral interventions, caregiver support, devices, and dietary supplements.

### Bias Assessment

The risk of bias was assessed in each of the 56 primary articles on AD by 2 reviewers (F.C.Q., M.T.F.) using the Cochrane bias assessment.^21^ Details are given in eAppendix 1 in the Supplement.

### Data Extraction

For each study included in this review, 1 reviewer (J.M.) extracted the required information into a prespecified extraction table according to the protocol. In brief, this included basic information on the trial, publication year, numbers of participants (men, women, and total), and a binary yes-or-no assessment of sex stratification in the methods, results, and protocol. The status of the drug (experimental or approved) was also recorded; we considered approved drugs as 1 of the 4 drugs currently in use for the treatment of AD (memantine, rivastigmine, donepezil, and galantamine). More details are available in eAppendix 1 in the Supplement.

### Statistical Analysis

We analyzed both primary and secondary articles using a specific rationale that avoided overfitting. Primary articles that included information about how many men and women were included in the study design were used to investigate the sex ratio in a trial overall, in prespecified subgroups, and across time. To study temporal trends in the reporting of sex in study results, we analyzed pooled primary and secondary articles together because secondary articles (with additional analysis of existing data) might have reported sex-stratified results.

A total of 56 primary articles for AD dementia and 8 for prodromal AD were analyzed separately per protocol; the analyses for prodromal AD did not find significant differences in sex ratios between subgroups, likely owing to low statistical power, and are not reported in this article. Pooling the results did not affect the overall conclusions of this study.

Within the primary articles, we examined sex ratios at baseline in prespecified subgroups (including approved vs experimental drugs, trial phase, and location) and the effect of prespecified variables (including the mean baseline Mini-Mental State Examination [MMSE] score, baseline age, trial duration in weeks, publication year, and year of trial start). Basic characteristics were calculated on a per-trial basis. If the mean value per trial was not given (eg, for age or MMSE), we calculated the weighted mean of the subgroups for which these data were available.

#### Sex Proportion

**Figure 2.**
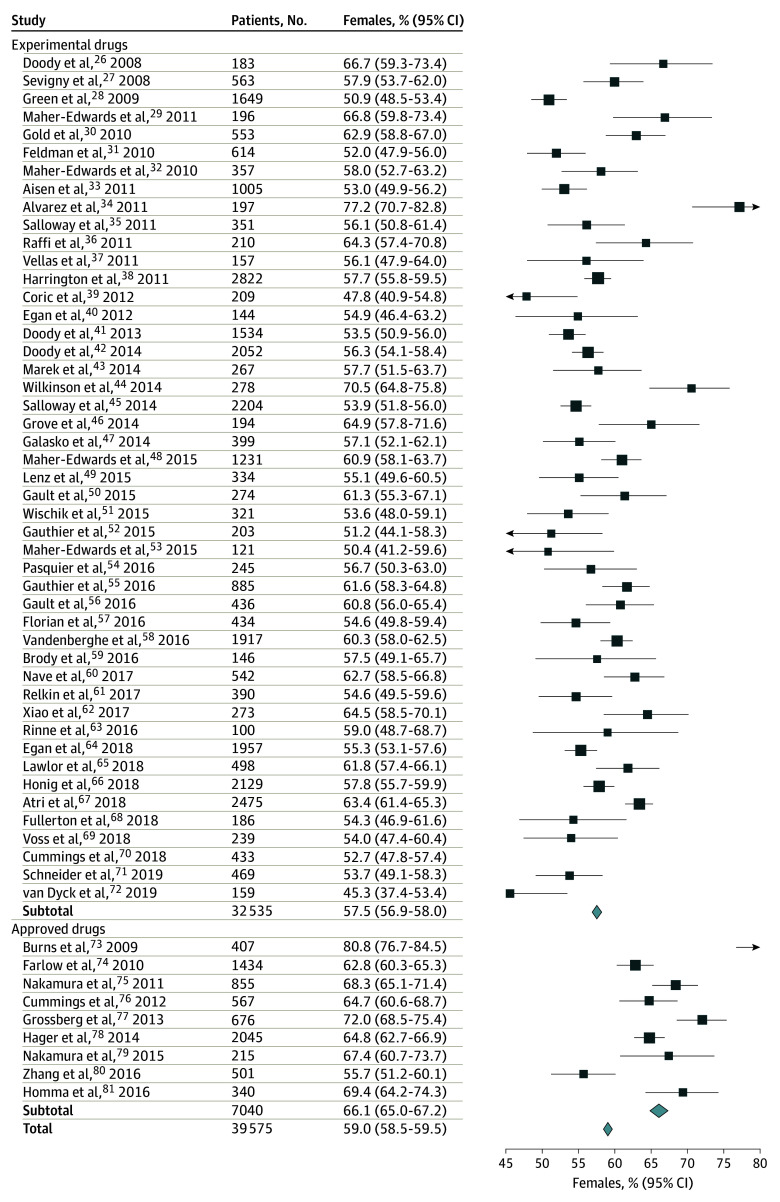
Proportion of Women in Primary Alzheimer Disease (AD) Articles Percentages were obtained by pooling raw data of all patients in 56 primary articles, defined as the chronologically first publication of the main results of a trial. Squares represent percentages, with horizontal lines indicating binomial 95% CIs. Diamonds represent pooled estimates, with points of the lines indicating 95% CIs. The marker size is proportional to the precision of the estimate.

An analysis of sex proportion was performed only for the 56 primary articles on AD. The total baseline percentage of women was calculated per trial arm and for the whole trial population by calculating the mean proportions of female individuals in each trial (ie, on a per-trial basis); in addition, we obtained similar proportions by pooling patients from all trials (Figure 2). We used Wilcoxon signed rank tests to compare the trial-based results with a fixed value of the proportion of women in the real-world population with AD based on published US^5^ (62.1%*)* and European^4^ (68.2%) statistics.

To assess the associations between prespecified variables, we used a multivariate mixed-effect logistic regression model of the probability that an enrolled patient was a woman. A logistic regression model was used because sex is a binary variable. We used 2 main models. Model 1 excluded location variables, and model 2 included all variables. To account for different sample sizes of the trials and intertrial variability, we added a random intercept at the trial level and estimated the corresponding variance parameter.^22,23^ Subsequently, a Spearman nonparametric correlation matrix was used to characterize the correlation between different trial characteristics. A series of pairwise comparisons was run between the coefficients of different locations using Bonferroni adjustment for multiple comparisons.

Pearson correlations were calculated to examine whether there was a temporal trend in the proportion of women; significance of the association was assessed using a 2-tailed Wald test. Correlations were weighted based on the total number of trial participants.

#### Sex-Stratified Data

Because RCT data may be subject to several analyses, resulting in multiple articles about the same trial, for this study, we used a pooled data set including both the primary and the secondary (later) articles. We calculated the percentages of articles that included a data analysis by sex in the study protocol, in the methods, and/or in the results sections of the article.

Temporal trends were calculated using logistic regression of the probability that the article reported sex-stratified results in the pooled data set of primary and secondary articles. All statistical analyses were conducted using R, version 3.6.2 (R Project for Statistical Computing).^24^ Unless otherwise specified, significance was set at 2-tailed *P* < .05.

## Results

### Basic Characteristics of Included Articles

A total of 1047 trials were identified on ClinicalTrials.gov (Figure 1). Among these, 409 published articles were found using PubMed, Google Scholar, Scopus, and article references; 70 articles were removed as duplicates and another 240 were excluded based on title and abstract screening.

By applying the predefined set of inclusion and exclusion criteria, we selected 77 articles, of which 64 were categorized as primary (56 on AD dementia and 8 on prodromal AD) and 13 as secondary (all on AD dementia). The most common reasons for excluding an article were population (ie, <100 participants) and study type (ie, not a randomized clinical trial). Agreement between reviewers was 95.6%.

The 56 selected primary articles on patients with AD dementia^25,26,27,28,29,30,31,32,33,34,35,36,37,38,39,40,41,42,43,44,45,46,47,48,49,50,51,52,53,54,55,56,57,58,59,60,61,62,63,64,65,66,67,68,69,70,71,72,73,74,75,76,77,78,79,80^ reported large phase 2 and 3 trials involving a median of 403.0 participants (interquartile range, 213.8-862.5 participants) with a mean (SD) age of 73.5 (2.5) years (Table 1). Nine articles (16.1%)^72,73,74,75,76,77,78,79,80^ reported results of approved drugs and 47 (83.9%)^25,26,27,28,29,30,31,32,33,34,35,36,37,38,39,40,41,42,43,44,45,46,47,48,49,50,51,52,53,54,55,56,57,58,59,60,61,62,63,64,65,66,67,68,69,70,71^ reported results of experimental drugs. Most articles (34 [60.7%])^25,26,28,29,31,32,33,35,37,38,39,40,41,42,43,45,46,47,48,49,50,52,53,56,59,60,62,65,67,71,72,75,79,80^ reported the sex ratio in the intention-to-treat population (Table 1). Basic information on trials and articles regarding prodromal AD^81,82,83,84,85,86,87,88^ and pooled trials and articles is available in eTables 2 and 3 in the Supplement. The references for the trials are available in eTable 4 of the Supplement, and a summary of the extracted data are available in eTable 5 in the Supplement.

**Table 1.** Basic Characteristics of Included Articles

Characteristic	Articles^a^
Articles on AD dementia, No.	56
Participants per article, median (IQR), No.	403.0 (213.8-862.5)
Sex, pooled No. (%)	
Women	23 348 (59.0)
Men	16 227 (41.0)
Age, mean (SD), y	73.5 (2.5)
Trial phase	
2	32 (57.1)
3	24 (42.9)
Year of publication, median (IQR)	2014.50 (2011.00-2016.00)
Year of trial start, median (IQR)	2008.00 (2006.75-2011.25)
Year of trial end, median (IQR)	2011.00 (2009.00-2014.00)
Trial duration, median (IQR), wk	25.0 (24.0-76.5)
Trial location	
Asia	6 (10.7)
Europe	6 (10.7)
North America	16 (28.6)
Worldwide	28 (50.0)
Trial population	
ITT	34 (60.7)
mITT	9 (16.1)
Safety	13 (23.2)
Severity of AD	
Mild to moderate	52 (92.9)
Severe	4 (7.1)
Approval status of drug	
Approved	9 (16.1)
Experimental	47 (83.9)
Mean MMSE score at baseline, median (IQR)^b^	19.16 (17.49-20.91)

Abbreviations: AD, Alzheimer disease; IQR, interquartile range; ITT, intention to treat; mITT, modified intention to treat; MMSE, Mini-Mental State Examination.

^a^ Data are presented as the number (percentage) of articles unless otherwise indicated. The mean (SD) is reported for normally distributed variables and the median (IQR) for non–normally distributed variables. Categorical variables are reported as the percentage of the total. All variables were assessed per trial as reported at baseline.

^b^ Data were available in 55 of the trials.

### Sex Proportion

In the 56 primary articles on AD dementia,^25,26,27,28,29,30,31,32,33,34,35,36,37,38,39,40,41,42,43,44,45,46,47,48,49,50,51,52,53,54,55,56,57,58,59,60,61,62,63,64,65,66,67,68,69,70,71,72,73,74,75,76,77,78,79,80^ the overall proportion of women was 59.0% (23 348 of 39 575 total participants) (Table 1 and Figure 2). In a preliminary data analysis, on a trial basis, the mean (SD) proportion of women in the trials for approved drugs^72,73,74,75,76,77,78,79,80^ was 67.3% (6.9%), whereas in trials for experimental medications,^25,26,27,28,29,30,31,32,33,34,35,36,37,38,39,40,41,42,43,44,45,46,47,48,49,50,51,52,53,54,55,56,57,58,59,60,61,62,63,64,65,66,67,68,69,70,71^ it was 57.9% (5.9%). The proportion of women in the experimental medications subgroup (57.9%; 95% CI, 55.8%-59.2%) was significantly different from the proportion of women in the population with AD in both in the US (62.1%; difference, −4.56% [95% CI, −6.29% to −2.87%]; *P* < .001) and Europe (68.2%; difference, −10.67% [95% CI, −12.39% to −8.97%]; *P* < .001).

In model 1 (Table 2), in which location variables were excluded, variables significantly associated with the probability that women were enrolled in a study included the status of the drug (approved vs experimental) and the severity of the participants’ AD (measured by baseline MMSE). Supporting the preliminary data analysis, trials involving drugs with approved status were associated with a higher probability of including women (odds ratio [OR], 1.26; 95% CI, 1.05-1.52; *P* = .02). However, we found a lower probability of women being included in trials with a higher mean baseline MMSE (OR, 0.98; 95% CI, 0.97-1.00; *P* = .02), indicating that trials including participants with more severe cases of AD were more likely to enroll women. The results were confirmed in pairwise comparisons (eTable 6 in the Supplement).

**Table 2.** Summary of Fixed Effects in Multivariate Mixed Effect Logistic Regression Models of the Probability That an Enrolled Trial Patient Was a Woman

Fixed effect	OR (95% CI)	*z* score	*P* value
Model 1^a^			
Intercept	1.51 (1.34-1.69)	6.74	<.001^b^
MMSE	0.98 (0.97-1.00)	−2.26	.02^c^
Age	1.02 (0.99-1.05)	1.22	.22
Year started	0.99 (0.96-1.03)	−0.47	.64
Year published	1.00 (0.96-1.03)	−0.31	.76
Status of drug (approved)	1.26 (1.05-1.52)	2.44	.02^c^
Trial duration	0.93 (0.83-1.05)	−1.16	.25
Model 2^d^			
Intercept	1.53 (1.37-1.71)	7.70	<.001^b^
MMSE	0.99 (0.97-1.00)	−1.79	.07
Age	1.03 (1.00-1.05)	1.83	.07
Year started	1.00 (0.97-1.03)	0.02	.98
Year published	0.98 (0.95-1.01)	−1.17	.24
Status of drug (approved)	1.10 (0.91-1.31)	0.98	.33
Trial duration	0.96 (0.86-1.07)	−0.74	.46
Location			
Asia	1.16 (0.94-1.42)	1.39	.16
Europe	1.26 (1.05-1.52)	2.45	.01^c^
North America	0.81 (0.71-0.93)	−3.10	.002^b^

Abbreviations: MMSE, Mini-Mental State Examination; OR, odds ratio.

^a^ Location excluded.

^b^ Significance level *P* = .01.

^c^ Significance level *P* = .05.

^d^ Location included.

When location was included in model 2, fewer associations were found (Table 2 and Figure 2).^25,26,27,28,29,30,31,32,33,34,35,36,37,38,39,40,41,42,43,44,45,46,47,48,49,50,51,52,53,54,55,56,57,58,59,60,61,62,63,64,65,66,67,68,69,70,71,72,73,74,75,76,77,78,79,80^ In model 2, location was the only factor significantly associated with inclusion of women in AD trials, with location in Europe associated with a higher probability that the trials included women (OR, 1.26; 95% CI, 1.05-1.52; *P* = .01) and location in North America associated with a lower probability (OR, 0.81; 95% CI, 0.71-0.93; *P* = .002) (Table 2; further results are shown in eTable 7 and location pairwise comparisons in eTable 8 in the Supplement).

Trial duration, mean baseline age of participants, publication year, and trial start year were not significantly associated with the probability that women were included, based on the results of either model. We did not find any significant temporal trend in the proportion of women included in AD trials over time, either by publication year or by trial start year (*R*, −0.04; 95% CI, −0.30 to 0.23; *P* = .79) (eFigure 1 in the Supplement). The variance parameter for the trial random effect was 0.036, confirming the presence of a nonnegligible degree of heterogeneity between trials.

### Reporting of Sex-Stratified Data and Its Temporal Trend

We investigated the proportion of primary articles that included sex stratification in the study protocol or methods or reported sex-stratified data in the results (eTable 9 in the Supplement). Most did not include sex-stratified data in the protocol, methods, or results.

Of the 56 AD dementia articles, we were able to identify a complete published protocol with a statistical analysis plan for only 17 (30.4%)^28,37,40,41,44,45,47,52,54,55,63,64,65,66,68,70,71^; of these, only 8 (47.1%)^40,41,54,55,63,65,68,70^ included a sex-specific data analysis in the protocol. Of the 56 total articles, 8 (14.3%)^27,49,54,55,56,64,67,68^ incorporated sex-specific data analysis in the methods section. Seven articles (12.5%)^27,49,55,56,64,67,68^ reported the results of such analysis, and 1 article^64^ showed a potential sex difference in efficacy that favored men, although no significance testing was conducted. No trials stratified trial arms by sex; the most common method of statistical analysis was a prespecified subgroup analysis.

To assess whether subsequent articles for a given RCT reported sex-stratified data, we also considered the secondary articles. We found that in this group of 13 articles,^89,90,91,92,93,94,95,96,97,98,99,100,101^ a sex-specific data analysis was present in 4 (30.8%).^91,92,99,100^

**Figure 3.**
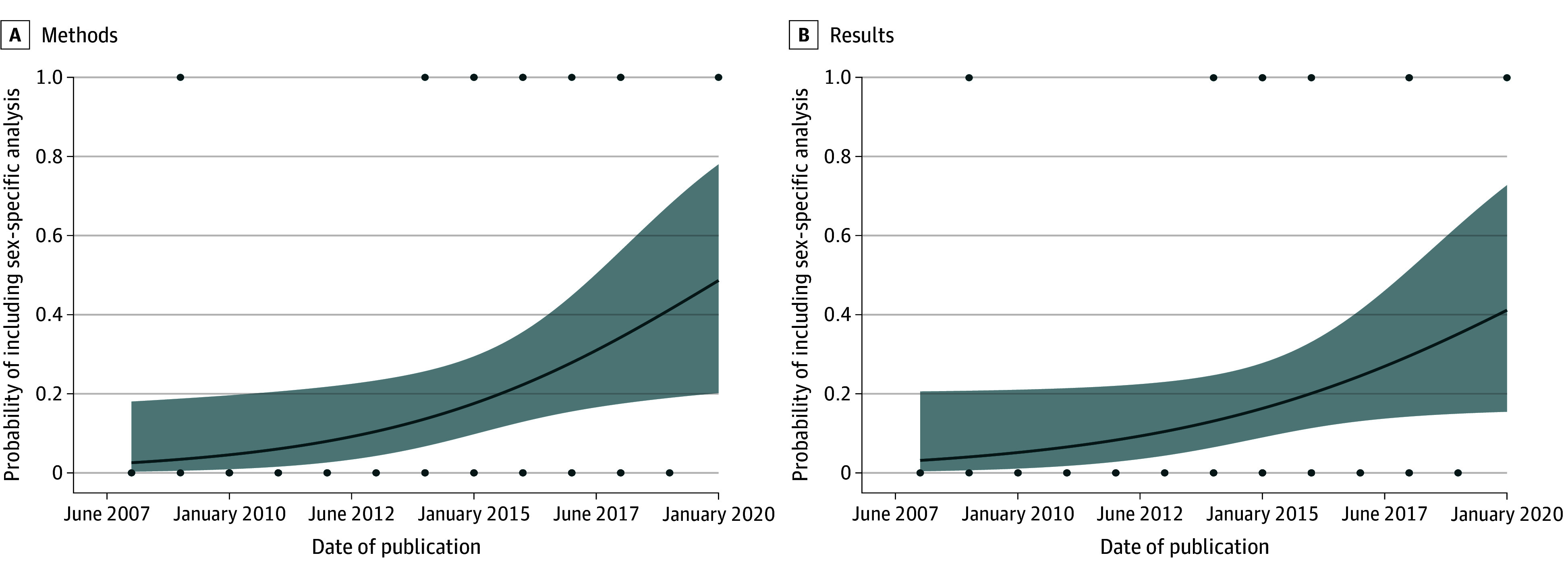
Temporal Trends in the Reporting of Sex-Stratification Analyses The trend was significant only for the methods (*R*, 0.30; 95% CI, 0.05-0.59; *P* = .03). The y-axis represents the probability of inclusion of a sex-specific analysis in a study, with 1 indicating that the study included a sex-specific analysis and 0 indicating that the study did not include a sex-specific analysis. Data markers indicate observed data points, and shading, the 95% CI.

Using a pooled data set from primary and secondary articles (Figure 3 and eFigure 2 in the Supplement), we found a statistically significant increasing temporal trend of articles that referenced a sex-specific data analysis in the methods (*R*, 0.30; 95% CI, 0.05-0.59; *P* = .03) and a similar trend for sex stratification in the results (*R*, 0.26; 95% CI, 0.01-0.55; *P* = .055). The results of the risk-of-bias analysis are provided in eAppendix 2 and eTable 10 in the Supplement.

## Discussion

In this systematic review and meta-analysis, of the 56 selected RCTs, 59.0% of the included participants were women, and 57.9% were women in the subgroup of trials of experimental drugs. Although this indicated greater trial enrollment of women compared with men, these numbers are significantly lower than the proportions of women reported in real-world populations with AD (68.2% in Europe and 62.1% in the US). This suggests that the enrollment of women in RCTs for AD could be further increased. Older women might be a particularly difficult group to enroll; their underrepresentation in RCTs is well-known in stroke research.^20^

To gain additional insights for the design of future RCTs, we analyzed factors associated with the probability of enrolling women in trials. A multivariate analysis revealed that trial duration was not associated with enrollment of women, whereas geographical and clinical factors were.

This study found that the probability that women were included in RCTs for AD was lower in RCTs in North America compared with other locations (eg, Europe). This observation, if confirmed, might indicate the need for region-specific strategies for enrollment of women in trials.

Aside from the location of the trials, drug status (approved vs experimental) was the factor most associated with differences in sex ratios. The RCTs for approved drugs had a significantly higher probability of including women than did RCTs for experimental drugs. The reasons for such differences remain to be elucidated; we are investigating the possibility that a higher ratio of women may be associated with trials in which a drug showed a significant clinical effect. In addition, we found that the probability of women’s inclusion was higher in trials involving more severe cases of AD, but this was not associated with age (Table 2); recruitment and retention of women in AD trials might therefore need to be tailored according to disease stage.

Of interest, we found that although an analysis of sex-based data was included in many available study protocols, the results of such analyses were not published in most cases. However, a temporal trend was found, indicating an increase in the inclusion of data analysis by sex in reports of AD trials.

The findings of this study may stimulate a global discussion on 3 important aspects associated with diversity in RCTs. First, when studying a multifactorial disease such as AD, properly representing the diverse patient population may be crucial in RCT design.^102^ Having a study population similar to the real-world one might be needed to detect relevant outcomes in a trial. For example, RCTs for migraine, a disease that largely affects women and for which several new drugs have been discovered, enroll more women than men, with proportions that reflect the expected real-world sex ratio.^103^ Of course, promoting women’s enrollment in RCTs for AD has to be weighed against the wider request by regulatory agencies for equality in RCT participation.

Second, participation of women and particularly older women in RCTs might be subject to specific challenges. When living alone, older women affected by AD or stroke might have a disadvantage in joining long and complex trials and might lack a caregiver to accompany them. Another possibility is that inclusion and exclusion criteria for RCTs—for instance, based on educational level—might unintentionally but systematically exclude more women than men.^104^

Third, the low frequency of sex-stratified results reported in articles is a call to action for better publishing practices. The data analysis revealed a low percentage of trials with complete protocols available (30.4%), a percentage that should increase for the sake of transparency. Describing sex-stratified data (even if no differences are found) should become a routine in clinical data publication, and it is also important for avoiding publication bias.

### Limitations

This study has limitations. First, as done in previous studies,^18,19,20^ we chose to use ClinicalTrials.gov as the primary source of RCT data. ClinicalTrials.gov allows registration of trials from all countries (exemplified by the different locations in the current data analyses). However, because only RCTs in the US are required to register at ClinicalTrials.gov, it is possible that this study’s data analysis was skewed toward RCTs conducted in the US. The highly selective inclusion and exclusion criteria also potentially led to exclusion of some relevant trials but enabled a more focused interpretation of results.

Another limitation is that owing to the exclusion of solely pharmacokinetic and safety trials from the data analysis, the potential sex differences in these aspects were not captured. It is well-known that drugs used in AD, such as antipsychotic medications, have different safety and pharmacokinetic profiles in men and women.^105^ Sex differences in adverse events have also been observed for rivastigmine^106^ and memantine.^107^ Therefore, further systematic exploration of sex differences in safety profiles is warranted.

In addition, some of the data analyses were based on imbalanced groups (for instance, analyses between trials of approved drugs [n = 9] vs trials of experimental drugs [n = 47]). Such imbalances potentially introduced a lack of statistical power for detecting differences. However, because the study’s approach was systematic, this was unlikely to be a source of bias that invalidated the results.

## Conclusions

In this systematic review and meta-analysis, the proportion of women in RCTs for AD, although higher than the proportion of men, was significantly lower than that in the general population. Only a small proportion of trials reported sex-stratified results. These findings support strategies to improve diversity in enrollment and data reporting in RCTs for AD.

## Notes

Supplement. **eAppendix 1.** Supplementary methods **eAppendix 2.** Supplementary results **eFigure 1.** Temporal trends in women’s representation in AD trials **eFigure 2.** Temporal trends in reporting of sex-stratified results by trial start year **eTable 1.** Inclusion and exclusion criteria according to PICOS **eTable 2.** Basic characteristics of included publications for prodromal AD, primary publications **eTable 3.** Basic characteristics of included publications: pooled AD dementia + prodromal AD, primary publications **eTable 4.** References for all included studies **eTable 5.** Overview of study details and extracted data **eTable 6.** Proportion of women in subgroups: nonparametric correlation matrix **eTable 7.** Multivariate mixed effect logistic regression model with probability of trial participant being a woman as a dependent variable, Model 3 (location only): summary of fixed effects **eTable 8.** Odds ratio of female enrollment by location: pairwise comparisons **eTable 9.** Reporting of sex-stratified data in primary studies (n=56) **eTable 10.** Bias assessment table
eReferences
