# Supplementary material for: Proportion of Women and Reporting of Outcomes by Sex in Clinical Trials for Alzheimer Disease: A Systematic Review and Meta-analysis
Source: JAMA Netw Open. 2021 Sep 13;4(9):e2124124. doi: 10.1001/jamanetworkopen.2021.24124 (PMC12243614; doi:10.1001/jamanetworkopen.2021.24124)
Supplement: Supplement. — eAppendix 1. Supplementary methods eAppendix 2. Supplementary results eFigure 1. Temporal trends in women’s representation in AD trials eFigure 2. Temporal trends in reporting of sex-stratified results by trial start year eTable 1. Inclusion and exclusion criteria according to PICOS eTable 2. Basic characteristics of included publications for prodromal AD, primary publications eTable 3. Basic characteristics of included publications: pooled AD dementia + prodromal AD, primary publications eTable 4. References for all included studies eTable 5. Overview of study details and extracted data eTable 6. Proportion of women in subgroups: nonparametric correlation matrix eTable 7. Multivariate mixed effect logistic regression model with probability of trial participant being a woman as a dependent variable, Model 3 (location only): summary of fixed effects eTable 8. Odds ratio of female enrollment by location: pairwise comparisons eTable 9. Reporting of sex-stratified data in primary studies (n=56) eTable 10. Bias assessment table eReferences [file jamanetwopen-e2124124-s001.pdf]

Supplementary Online Content

Martinkova J, Quevenco FC, Karcher H, et al. Proportion of women and reporting of outcomes by sex in clinical trials for Alzheimer disease: a systematic review and meta-analysis. *JAMA Netw Open*. 2021;4(9):e2124124. doi:10.1001/jamanetworkopen.2021.24124

eAppendix 1. Supplementary methods
eAppendix 2. Supplementary results
eFigure 1. Temporal trends in women's representation in AD trials
eFigure 2. Temporal trends in reporting of sex-stratified results by trial start year
eTable 1. Inclusion and exclusion criteria according to PICOS
eTable 2. Basic characteristics of included publications for prodromal AD, primary publications
eTable 3. Basic characteristics of included publications: pooled AD dementia + prodromal AD, primary publications
eTable 4. References for all included studies
eTable 5. Overview of study details and extracted data
eTable 6. Proportion of women in subgroups: nonparametric correlation matrix
eTable 7. Multivariate mixed effect logistic regression model with probability of trial participant being a woman as a dependent variable, Model 3 (location only): summary of fixed effects
eTable 8. Odds ratio of female enrollment by location: pairwise comparisons
eTable 9. Reporting of sex-stratified data in primary studies (n=56)
eTable 10. Bias assessment table
eReferences

This supplementary material has been provided by the authors to give readers additional information about their work.

© 2021 Martinkova J et al. *JAMA Network Open*.

eAppendix 1. Supplementary Methods.

Definition of sex and gender
Sex refers to the biological characteristics arising from the expression of sex chromosomes (XX for female and XY for male) as well as the effects of exogenous gonadal hormones. Gender refers to the socially-driven definition of being a man and a woman in a particular society[1].
In current RCTs, whether the patient is man or woman is either self-reported or assessed by a clinician. Therefore, we chose to use the term 'sex' throughout the manuscript referring to self-reported sex.

Search strategy and selection
The literature review search was conducted in two stages to minimise publication omissions. In the first stage, the following sources were sequentially searched, each by one reviewer: PubMed (FCQ) on 4th September, using the national clinical trial identifier, AND/OR principal investigator AND/OR trial name. Google Scholar (JM) and Scopus (JM) were searched from 5th September to 31st October 2019, using the national clinical trial identifier, and where available, principal investigator and trial name. Internal analyses revealed that the primary articles on several clinical trials we had selected were not included in these search results. These publications were not available on Pubmed, but were found in Google Scholar. Therefore, a second additional search was conducted by JM from 15th April to 31st May 2020 on Google Scholar and Scopus only, specifically for the clinical trials where no article had been found. Here, only the national clinical identifier was used as search criteria since we observed that this maximized search yield, i.e., the strategy enabled missing primary publications to be included. While the second search was not included in the original protocol, it did not significantly change the results of the study and allowed for inclusion of more publications than the original search.

Bias assessment
The following risk domains were evaluated: random sequence generation (selection bias); allocation concealment (selection bias); blinding of participants and personnel (performance bias); blinding of outcome assessment (detection bias); incomplete outcome data (attrition bias); selective reporting (reporting bias); other sources of bias. Each study was rated by two independent researchers (M.T.F. and F.C.Q.) as having low risk of bias (LR), high risk of bias (HR), or unclear risk of bias because of not enough information (NI). Disagreements between the authors were low and were resolved by authors' consensus.

Data extraction
From each trial we extracted: title, authors, date of publications, NCT study identifier, trial start and end date year, and where available, trial sponsor, number and age of subjects enrolled; whether the publication reported the distribution of sex in the study population, the number of subjects of each sex, the population for which sex distribution was reported; whether the study protocol had a pre-specified analysis by sex in Methods, whether sex-stratified results were reported in publication Results; disease severity in study subjects. Additionally, trial location (simplified to continents, we defined as 'worldwide' studies that

© 2021 Martinkova J et al. *JAMA Network Open*.

were located on two or more continents), availability of study protocol, sex distribution in control and verum treatment groups, number of verum groups, control type, whether any analysis where the effect of sex could be determined was reported, reported sex difference, trial duration in weeks, mean baseline MMSE per trial and binarized result of the trial (verum effective/not effective) were also recorded.

Analysis tools
All analyses were conducted in R,[2] some utilizing the weights package[3] and the binom package.[4] Figures were created using the ggplot2[5] and forestplot[6] packages.
Table 1 and eTables 2 and 3 were created using the tableone package[7].

The planned subgroup analyses based on severity categories reported in the original publications produced extremely unbalanced subgroups (n=4 in the 'severe' category and n=52 in the 'mild to moderate' category) and were therefore not further examined. Instead, we utilized mean baseline MMSE per trial as a continuous predictor variable.

eAppendix 2. Supplementary results.

An analysis of the temporal trend of female proportion by phase of study, for both treatment groups (placebo/verum), and for the North America subgroup, was performed and found not significant.
We explored the correlations between different study-level variables and between study-level variables and proportion of women enrolled by a series of multivariate analyses.
eTable 6 displays Spearman non-parametric correlation matrix of severity, age, study start date, study publication date, approved versus experimental drug, location variables and proportion of women, with tests on the coefficients.
The proportion of women correlated negatively with mean baseline MMSE per trial ((rho=–0.62, p<0.001), indicating a higher proportion of women in trials with lower participant MMSE, i.e. higher severity.
There was a positive correlation with approved drug status (rho=0.47, p<0.001). All included location variables have significant correlation with the proportion of women, which is negative in North America (rho=–0.49, p<0.001) and positive in Asia and Europe (rho=0.34, p=0.01 and rho=0.28, p=0.04, respectively).
Year of trial start and publication show a trivial significant correlation, while severity decreases with age and is lower in trials with experimental drugs. Finally, later published trials enrolled on average younger patients.

Risk of bias
We performed a risk of bias assessment according to the Cochrane bias assessment guide (eTable 10). As all publications were double-blind RCTs, selection bias was overall low, even though several publications failed to detail procedure for allocation concealment. In a few publications, we detected elements, which might have potentially caused the breaking of blind by participants (for instance, obvious side effects in *verum* vs placebo, such as ARIA) or by the experimenter (detection bias). Incomplete outcome data, with imputation methods missing or unclear, and high number of dropouts was found in 10 publications. Finally, the majority of publications did not have a public protocol available, hence we could not evaluate reporting bias.

© 2021 Martinkova J et al. *JAMA Network Open*.

Supplementary Figures

eFigure 1: Temporal trends in women's representation in AD trials.

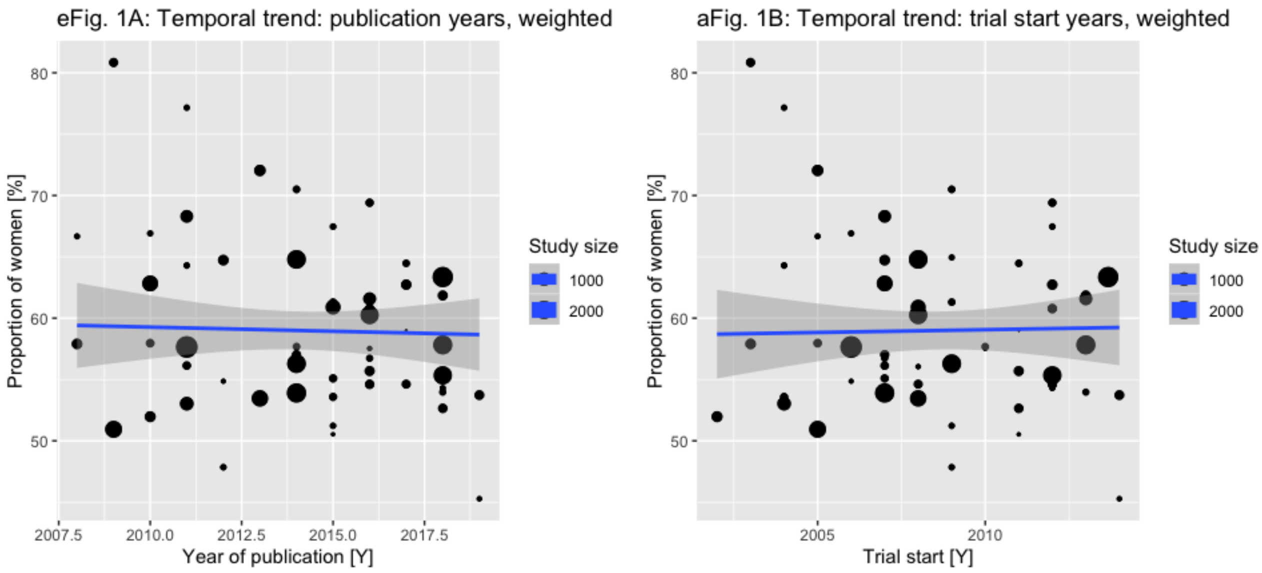

eFigure 1: Temporal trends in women's representation in AD trials. Proportion of women in AD CTs per publication year (of primary publications) was analyzed by Pearson correlation. When weighted by total population per trial, both year of publication and year of trial start show non-significant results. Shading indicates 95% confidence interval.

eFigure 2: Temporal trends in reporting of sex-stratified results by trial start year

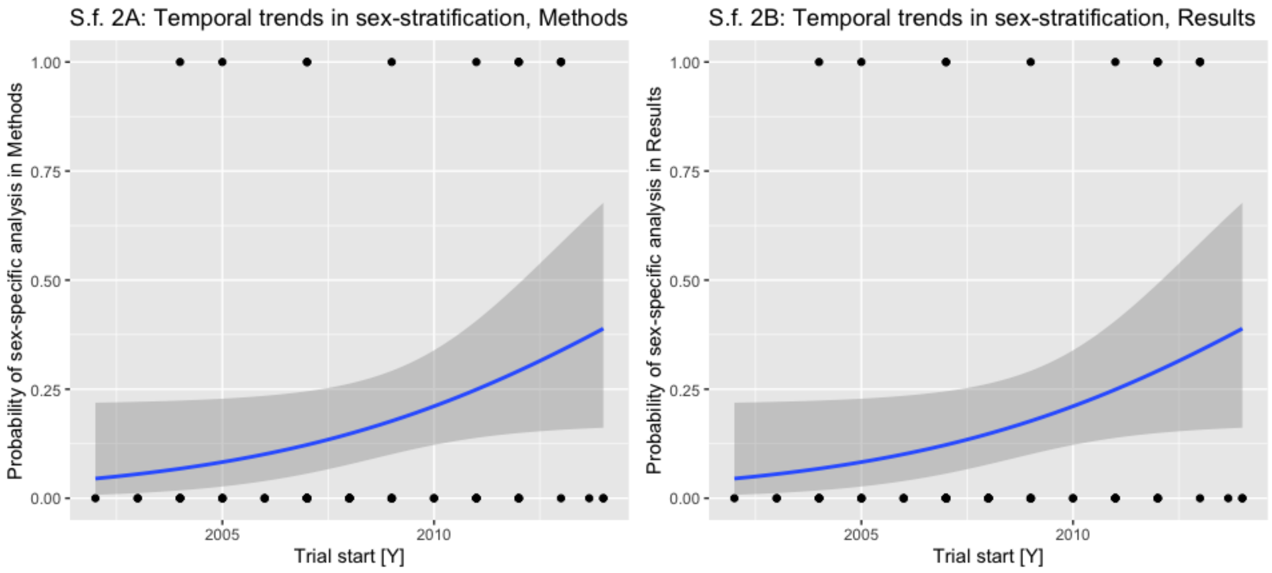

eFigure 2: Temporal trends in reporting of sex-stratified results by trial start year. The diagram shows a positive trend in the number of publications (Y axis) reporting sex-stratified analysis in the Methods section as measured by trial start year, eFigure 2B shows a similar

© 2021 Martinkova J et al. *JAMA Network Open*.

trend for reporting these results in the Results section. Shading indicates 95% confidence interval, y-axis: 1: included sex stratification, 0: did not include it.

© 2021 Martinkova J et al. *JAMA Network Open*.

Supplementary Tables

eTable 1: Inclusion and exclusion criteria according to PICOS

PICOS dimension	Inclusion criteria	Exclusion criteria
Population	Study with patients diagnosed with dementia due to AD (mild, moderate, or advanced)<br><br>MCI due to AD (based on biomarker evidence)<br><br>Combined studies of MCI due to AD and AD patients, if data are reported separately and not pooled<br><br>Study with both men and women enrolled<br><br>Studies with at least 100 participants	Combined studies which examined AD dementia and other dementia types (i.e. vascular)<br><br>Studies which used healthy volunteers or other dementias.<br><br>Down Syndrome and familial AD.<br><br>Mild cognitive impairment not due to AD.<br><br>Studies with only men or only women<br><br>Studies with less than 100 participants<br><br>Studies on AD patients focusing on neuropsychiatric symptoms such as agitation, psychosis, insomnia
Intervention	Pharmacological, biological or genetic agents<br>Herbal extracts	Behavioral studies, studies on caregivers, devices, dietary supplements
Comparator	Any comparator. Placebo controlled, gold standard controlled	Studies examining the effect of the discontinuation of a drug
Outcome	Clinical efficacy on cognition and memory; changes in AD ATN biomarker status	Safety, tolerability, bioavailability
Study type	RCT, phase 1, 2 and 3	Not randomized trials

eTable 2: Basic characteristics of included publications for prodromal AD, primary publications

© 2021 Martinkova J et al. *JAMA Network Open*.

Basic characteristics of included publications: prodromal AD, primary publications		
n	8	
Total N of subjects (median [IQR])	297.50 [153.75, 538.25]	
Sex, pooled [%]	Women	1392 (48.1%)
	Men	1500 (51.9%)
Average age [years] (mean (SD))	71.73 (1.10)	
Trial phase (%)	1	1 (12.5)
	2	5 (62.5)
	3	2 (25.0)
Year of publication (median [IQR])	2016.50 [2015.00, 2018.25]	
Year of trial start (median [IQR])	2011.00 [2009.75, 2013.50]	
Year of trial end (median [IQR])	2017.00 [2013.00, 2018.25]	
Trial duration [weeks] (median [IQR])	59.5 [15.75, 104.00]	
Trial locations (%)	Asia	0
	Europe	2 (25.0)
	North America	2 (25.0)
	Worldwide	4 (50.0)
Population (%)	ITT	7 (87.5)
	mITT	0
	Safety	1 (12.5)
Experimental/approved (%)	Approved	0
	Experimental	8 (100)
Lowest MMSE included (median [IQR])	22.50 [19.50, 24.00]	
Highest MMSE included (median [IQR])	30.00 [30.00, 30.00]	

© 2021 Martinkova J et al. *JAMA Network Open*.

Basic characteristics of included publications: prodromal AD, primary publications	
Mean MMSE at baseline* (median [IQR])	25.35 [24.40, 26.17]

eTable 2: Basic characteristics of included publications for prodromal AD, primary publications. Reported in mean (SD) for normally distributed variables and in median [IQR] for non-normally distributed variables, categorical variables reported in % of total. All variables per trial level, as reported at baseline.
* where available, n = 6
MMSE = mini mental state examination, IQR = interquartile range, SD = standard deviation, ITT = intention to treat, mITT = modified intention to treat

eTable 3: Basic characteristics of included publications: pooled AD dementia + prodromal AD, primary publications

Basic characteristics of included publications: pooled AD dementia + prodromal AD, primary publications		
n	64	
Total N of subjects (median [IQR])	394.50 [209.75, 811.50]	
Sex, pooled [%]	Women	24 740 (58.3%)
	Men	17 727 (41.7%)
Average age [years] (mean (SD))	73.26 (2.43)	
Trial phase (%)	1	1 (1.6)
	2	37 (57.8)
	3	26 (40.6)
Year of publication (median [IQR])	2015.00 [2011.75, 2017.00]	
Year of trial start (median [IQR])	2009.00 [2007.00, 2012.00]	
Year of trial end (median [IQR])	2012.00 [2010.00, 2015.00]	
Trial duration [weeks] (median [IQR])	26.00 [24.00, 78.00]	
Trial locations (%)	Asia	6 (9.4)
	Europe	8 (12.5)

© 2021 Martinkova J et al. *JAMA Network Open*.

Basic characteristics of included publications: pooled AD dementia + prodromal AD, primary publications		
	North America	18 (28.1)
	Worldwide	32 (50.0)
Population (%)	ITT	41 (64.1)
	mITT	9 (14.1)
	Safety	14 (21.9)
Severity (%)	Prodromal included	8 (12.5)
	Mild to moderate	52 (81.2)
	Severe included	4 (6.2)
Experimental/approved (%)	Approved	9 (14.1)
	Experimental	55 (85.9)
Lowest MMSE included (median [IQR])	12.00 [10.00, 16.00]	
Highest MMSE included (median [IQR])	26.00 [23.75, 26.00]	
Mean MMSE at baseline* (median [IQR])	19.22 [17.49, 21.37]	

eTable 3: Basic characteristics of included publications: pooled AD dementia + prodromal AD, primary publications. Reported in mean (SD) for normally distributed variables and in median [IQR] for non-normally distributed variables, categorical variables reported in % of total. All variables per trial level, as reported at baseline.
* where available, n = 61
MMSE = mini mental state examination, IQR = interquartile range, SD = standard deviation, ITT = intention to treat, mITT = modified intention to treat

eTable 4: References for all included studies

Trial ID	First author	Publication year	Publication title
NCT01739348	Egan	2018	Randomized Trial of Verubecestat for Mild-to-Moderate Alzheimer's Disease
NCT02079909	Schneider	2019	Safety and Efficacy of Edonerpic Maleate for Patients With Mild to Moderate Alzheimer Disease: A Phase 2 Randomized Clinical Trial

© 2021 Martinkova J et al. *JAMA Network Open*.

Trial ID	First author	Publication year	Publication title
NCT00710684	Maher-Edwards	2015	Two randomized controlled trials of SB742457 in mild-to-moderate Alzheimer's disease
NCT00555204	Lenz	2015	Adaptive, Dose-finding Phase 2 Trial Evaluating the Safety and Efficacy of ABT-089 in Mild to Moderate Alzheimer Disease
NCT00478205	Farlow	2010	Effectiveness and Tolerability of High-Dose (23 mg/d) Versus Standard-Dose (10 mg/d) Donepezil in Moderate to Severe Alzheimer's Disease: A 24-Week, Randomized, Double-Blind Study
NCT00478205	Ferris	2011	Analyzing the impact of 23 mg/day donepezil on language dysfunction in moderate to severe Alzheimer's disease
NCT00478205	Doody	2012	Efficacy and Safety of Donepezil 23 mg versus Donepezil 10 mg for Moderate-to-Severe Alzheimer's Disease: A Subgroup Analysis in Patients Already Taking or Not Taking Concomitant Memantine
NCT00478205	Salloway	2012	Subgroup Analysis of US and Non-US Patients in a Global Study of High-Dose Donepezil (23 mg) in Moderate and Severe Alzheimer's Disease
NCT00478205	Schmitt	2013	Evaluation of an 8-item Severe Impairment Battery (SIB-8) versus the full SIB in moderate to severe Alzheimer's disease patients participating in a donepezil study
NCT00377715	Doody	2008	Effect of dimebon on cognition, activities of daily living, behaviour, and global function in patients with mild-to-moderate Alzheimer's disease: a randomised, double-blind, placebo-controlled study
NCT00479557, NCT00498602	Pasquier	2016	Two Phase 2 Multiple Ascending–Dose Studies of Vanutide Cridificar (ACC-001) and QS-21 Adjuvant in Mild-to-Moderate Alzheimer's Disease
NCT02017340	Lawlor	2018	Nilvadipine in mild to moderate Alzheimer disease: A randomised controlled trial
NCT01689246	Gauthier	2016	Efficacy and safety of tau-aggregation inhibitor therapy in patients with mild or moderate Alzheimer's disease: a randomised, controlled, double-blind, parallel-arm, phase 3 trial
NCT02240693, NCT02337907	Frölich	2019	Evaluation of the efficacy, safety and tolerability of orally administered BI 409306, a novel phosphodiesterase type 9 inhibitor, in two randomised controlled phase II studies in patients with prodromal and mild Alzheimer's disease
NCT02389413	Scheltens	2018	Safety, tolerability and efficacy of the glutaminyl cyclase inhibitor PQ912 in Alzheimer's disease: results of a randomized, double-blind, placebo-controlled phase 2a study
NCT00679627	Hager	2014	Effects of galantamine in a 2-year, randomized, placebo-controlled study in Alzheimer's disease
NCT00679627	Hager	2016	Effect of concomitant use of memantine on mortality and efficacy outcomes of galantamine-treated patients with Alzheimer's disease: post-hoc analysis of a randomized placebo-controlled study
NCT02167256	van Dyck	2019	Effect of AZD0530 on Cerebral Metabolic Decline in Alzheimer Disease
NCT01900665	Honig	2018	Trial of Solanezumab for Mild Dementia Due to Alzheimer's Disease
NCT01955161, NCT02006641, NCT02006654	Atri	2018	Effect of Idalopirdine as Adjunct to Cholinesterase Inhibitors on Change in Cognition in Patients With Alzheimer Disease
NCT00348192	Maher-Edwards	2010	SB-742457 and donepezil in Alzheimer disease: a randomized, placebo-controlled study

© 2021 Martinkova J et al. *JAMA Network Open*.

Trial ID	First author	Publication year	Publication title
NCT01676935	Gault	2016	ABT-126 monotherapy in mild-to-moderate Alzheimer's dementia: randomized doubleblind, placebo and active controlled adaptive trial and open-label extension
NCT01549834	Florian	2016	Efficacy and Safety of ABT-126 in Subjects with Mild-to-Moderate Alzheimer's Disease on Stable Doses of Acetylcholinesterase Inhibitors: A Randomized, Double-Blind, Placebo-Controlled Study
NCT01677754	Nave	2017	Sembragiline in Moderate Alzheimer's Disease: Results of a Randomized, Double-Blind, Placebo-Controlled Phase II Trial (MAyflOwer RoAD)
NCT01677572	Sevigny	2016	The antibody aducanumab reduces Aβ plaques in Alzheimer's disease
NCT01712074	Fullerton	2018	A Phase 2 clinical trial of PF-05212377 (SAM-760) in subjects with mild to moderate Alzheimer's disease with existing neuropsychiatric symptoms on a stable daily dose of donepezil
NCT00905372, NCT00904683	Doody	2014	Phase 3 Trials of Solanezumab for Mild-to-Moderate Alzheimer's Disease
NCT01137526	Marek	2014	Efficacy and safety evaluation of HSD-1 inhibitor ABT-384 in Alzheimer's disease
NCT01224106	Ostrowiczki	2017	A phase III randomized trial of gantenerumab in prodromal Alzheimer's disease
NCT01019421	Wilkinson	2014	Safety and efficacy of idalopirdine, a 5-HT6 receptor antagonist, in patients with moderate Alzheimer's disease (LADDER): a randomised, double-blind, placebo-controlled phase 2 trial
NCT00818662	Relkin	2017	A phase 3 trial of IV immunoglobulin for Alzheimer disease
NCT00948909	Gault	2015	A phase 2 randomized, controlled trial of the a7 agonist ABT-126 in mild-to-moderate Alzheimer's dementia
NCT00911807	Alvarez	2011	Combination Treatment in Alzheimer's Disease: Results of a Randomized, Controlled Trial with Cerebrolysin and Donepezil
NCT00594568	Doody	2013	A Phase 3 Trial of Semagacestat for Treatment of Alzheimer's Disease
NCT00594568	Doody	2015	Peripheral and central effects of γ-secretase inhibition by semagacestat in Alzheimer's disease
NCT00890890	Coric	2015	Targeting Prodromal Alzheimer Disease With Avagacestat: A Randomized Clinical Trial
NCT00676143, NCT00667810	Vandenberghe	2016	Bapineuzumab for mild to moderate Alzheimer's disease in two global, randomized, phase 3 trials
NCT00428090	Gold	2010	Rosiglitazone Monotherapy in Mild-to-Moderate Alzheimer's Disease: Results from a Randomized, Double-Blind, Placebo-Controlled Phase III Study
NCT00810147	Coric	2012	Safety and Tolerability of the -Secretase Inhibitor Avagacestat in a Phase 2 Study of Mild to Moderate Alzheimer Disease
NCT00575055, NCT00574132	Salloway	2014	Two Phase 3 Trials of Bapineuzumab in Mild-to-Moderate Alzheimer's Disease
NCT00575055	Liu	2015	Amyloid-b 11C-PiB-PET imaging results from 2 randomized bapineuzumab phase 3 AD trials
NCT00575055	Samtani	2015	Alzheimer's disease assessment scale-cognitive 11-item progression model in mild-to-moderate Alzheimer's disease trials of bapineuzumab
NCT00420420	Egan	2012	Pilot Randomized Controlled Study of a Histamine Receptor Inverse Agonist in the Symptomatic Treatment of AD
NCT00074529	Sevigny	2008	Growth hormone secretagogue MK-677 : No clinical effect on AD progression in a randomized trial
NCT00506415	Cummings	2012	Randomized, Double-Blind, Parallel-Group, 48-Week Study for Efficacy and Safety of a Higher-Dose Rivastigmine Patch (15 vs. 10 cm2) in Alzheimer's Disease

© 2021 Martinkova J et al. *JAMA Network Open*.

Trial ID	First author	Publication year	Publication title
NCT00506415	Molinuevo	2015	Responder analysis of a randomized comparison of the 13.3 mg/24 h and 9.5 mg/24 h rivastigmine patch
NCT00216593	Burns	2009	Safety and efficacy of galantamine (Reminyl) in severe Alzheimer's disease (the SERAD study): a randomised, placebo-controlled, double-blind trial
NCT01953601	Egan	2019	Randomized Trial of Verubecestat for Prodromal Alzheimer's Disease
NCT00322153	Grossberg	2013	The Safety, Tolerability, and Efficacy of Once-Daily Memantine (28 mg): A Multinational, Randomized, Double-Blind, Placebo-Controlled Trial in Patients with Moderate-to-Severe Alzheimer's Disease Taking Cholinesterase Inhibitors
NCT00322153	Grossberg	2018	Memantine ER Maintains Patient Response in Moderate to Severe Alzheimer's Disease Post Hoc Analyses From a Randomized, Controlled, Clinical Trial of Patients Treated With Cholinesterase Inhibitors
NCT00105547	Green	2009	Effect of Tarenflurbil on Cognitive Decline and Activities of Daily Living in Patients With Mild Alzheimer Disease: A Randomized Controlled Trial
NCT00515333	Wischik	2015	Tau Aggregation Inhibitor Therapy: An Exploratory Phase 2 Study in Mild or Moderate Alzheimer's Disease
NCT01569516	Xiao	2017	Efficacy and safety of a novel acetylcholinesterase inhibitor octohydroaminoacridine in mild-tomoderate Alzheimer's disease: a Phase II multicenter randomised controlled trial
NCT00423085	Nakamura	2011	A 24-Week, Randomized, Double-Blind, Placebo-Controlled Study to Evaluate the Efficacy, Safety and Tolerability of the Rivastigmine Patch in Japanese Patients with Alzheimer's Disease
NCT00842816	Gauthier	2015	Effects of the Acetylcholine Release Agent ST101 with Donepezil in Alzheimer's Disease: A Randomized Phase 2 Study
NCT01009255	Grove	2014	A Randomized, Double-Blind, Placebo-Controlled, 16-Week Study of the H3 Receptor Antagonist, GSK239512 as a Monotherapy in Subjects with Mild-to-Moderate Alzheimer's Disease
NCT01852110	Voss	2018	Randomized, controlled, proof-of-concept trial of MK-7622 in Alzheimer's disease
NCT01324518	Rinne	2017	Tolerability of ORM-12741 and effects on episodic memory in patients with Alzheimer's disease
NCT01399125	Zhang	2016	Rivastigmine Patch in Chinese Patients with Probable Alzheimer's disease: A 24-week, Randomized, Double-Blind Parallel-Group Study Comparing Rivastigmine Patch (9.5 mg/24 h) with Capsule (6 mg Twice Daily)
NCT00568776	Salloway	2011	A phase 2 randomized trial of ELND005, scyllo-inositol, in mild to moderate Alzheimer disease
NCT00566397	Galasko	2014	Clinical trial of an inhibitor of RAGE-Ab interactions in Alzheimer disease
NCT01117818	Schneeberger	2015	Results from a Phase II Study to Assess the Clinical and Immunological Activity of AFFITOPE® AD02 in Patients with Early Alzheimer's Disease
NCT00083590	Rafii	2011	A phase II trial of huperzine A in mild to moderate Alzheimer disease
NCT01428453	Maher-Edwards	2015	A 24-week study to evaluate the effect of rilapladib on cognition and cerebrospinal fluid biomarkers of Alzheimer's disease
NCT00438568	Craft	2012	Intranasal Insulin Therapy for Alzheimer Disease and Amnestic Mild Cognitive Impairment
NCT00814801	Ohnishi	2014	The Prediction of Response to Galantamine Treatment in Patients with Mild to Moderate Alzheimer's Disease

© 2021 Martinkova J et al. *JAMA Network Open*.

Trial ID	First author	Publication year	Publication title
NCT01614886	Nakamura	2015	A 24-Week, Randomized, Controlled Study to Evaluate the Tolerability, Safety and Efficacy of 2 Different Titration Schemes of the Rivastigmine Patch in Japanese Patients with Mild to Moderate Alzheimer's Disease
NCT00880412	Vellas	2011	EHT0202 in Alzheimer's Disease: A 3-Month, Randomized, Placebo-Controlled, Double-Blind Study
NCT00053599	Feldman	2010	Randomized controlled trial of atorvastatin in mild to moderate Alzheimer disease
NCT00224497	Maher-Edwards	2010	Double-Blind, Controlled Phase II Study of a 5-HT6 Receptor Antagonist, SB-742457, in Alzheimer's Disease
NCT01343966	Cummings	2018	ABBY: A phase 2 randomized trial of crenezumab in mild to moderate Alzheimer disease
NCT01343966, NCT01397578, NCT02353598	Yoshida	2020	Pharmacokinetics and pharmacodynamic effect of crenezumab on plasma and cerebrospinal fluid beta-amyloid in patients with mild-to-moderate Alzheimer's disease
NCT01254773	Brody	2016	A Phase II, Randomized, Double-Blind, Placebo-Controlled Study of Safety, Pharmacokinetics, and Biomarker Results of Subcutaneous Bapineuzumab in Patients with mild to moderate Alzheimer's disease
NCT00348140	Harrington	2011	Rosiglitazone Does Not Improve Cognition or Global Function when Used as Adjunctive Therapy to AChE Inhibitors in Mild-to-Moderate Alzheimer's Disease: Two Phase 3 Studies
NCT00088673	Aisen	2011	Tramiprosate in mild-to-moderate Alzheimer's disease – a randomized, double-blind, placebo-controlled, multi-centre study (the Alphase Study)
NCT00088673	Abushakra	2017	Clinical Effects of Tramiprosate in APOE4/4 Homozygous Patients with Mild Alzheimer's Disease Suggest Disease Modification Potential
NCT01539031	Homma	2016	Efficacy and Safety of Sustained Release Donepezil High Dose versus Immediate Release Donepezil Standard Dose in Japanese Patients with Severe Alzheimer's Disease: A Randomized, Double-Blind Trial

eTable 5: Overview of study details and extracted data

Reference	Designation	Trial start	Trial duration [weeks]	Trial participants, N	Women, N	Women [%]	Location	Approved/experimental	Mean baseline MMSE	Mean baseline age [years]
Egan, 2018[8]	primary AD	2012	78	1957	1083	55.34	WW	experimental	NA	71.83
Schneider, 2019[9]	primary AD	2014	52	469	252	53.73	NoA	experimental	18.27	71.83
Maher-Edwards, 2015[10]	primary AD	2008	35	1231	750	60.90	WW	experimental	18.60	73.07
Lenz, 2015[11]	primary AD	2007	12	334	184	55.09	NoA	experimental	20.70	75.59
Farlow, 2010[12]	primary AD	2007	24	1434	901	62.83	WW	approved	13.10	73.87

© 2021 Martinkova J et al. *JAMA Network Open*.

Reference	Designation	Trial start	Trial duration [weeks]	Trial participants, N	Women, N	Women [%]	Location	Approved/ experimental	Mean baseline MMSE	Mean baseline age [years]
Doody, 2008[13]	primary AD	2005	26	183	122	66.67	AS	experimental	18.51	68.24
Pasquier, 2016[14]	primary AD	2007	104	245	139	56.73	WW	experimental	21.40	69.10
Lawlor, 2018[15]	primary AD	2013	78	498	308	61.85	EU	experimental	20.40	72.95
Gauthier, 2016[16]	primary AD	2013	65	885	545	61.58	WW	experimental	18.60	70.60
Hager, 2014[17]	primary AD	2008	104	2045	1325	64.79	EU	approved	19.00	73.00
van Dyck, 2019[18]	primary AD	2014	52	159	72	45.28	NoA	experimental	22.47	71.00
Honig, 2018[19]	primary AD	2013	80	2129	1231	57.82	WW	experimental	22.70	73.00
Atri, 2018[20]	primary AD	2014	24	2475	1568	63.35	WW	experimental	17.33	74.19
Maher-Edwards, 2010[21]	primary AD	2006	24	196	131	66.91	WW	experimental	18.99	71.20
Gault, 2016[22]	primary AD	2012	24	436	265	60.78	WW	experimental	18.90	74.20
Florian, 2016[23]	primary AD	2012	24	434	237	54.61	WW	experimental	18.93	75.10
Nave, 2017[24]	primary AD	2012	52	542	340	62.73	WW	experimental	17.03	73.03
Fullerton, 2018[25]	primary AD	2012	18	186	101	54.30	WW	experimental	19.65	75.95
Doody, 2014[26]	primary AD	2009	80	2052	1155	56.29	WW	experimental	21.00	73.56
Marek, 2014[27]	primary AD	2010	12	267	154	57.68	WW	experimental	19.20	72.00
Wilkinson, 2014[28]	primary AD	2009	24	278	196	70.50	WW	experimental	17.00	74.48
Relkin, 2017[29]	primary AD	2008	78	390	213	54.62	NoA	experimental	21.30	70.30
Gault, 2015[30]	primary AD	2009	12	274	168	61.31	WW	experimental	19.10	73.90
Alvarez, 2011[31]	primary AD	2004	28	197	152	77.16	EU	experimental	17.50	75.20

© 2021 Martinkova J et al. *JAMA Network Open*.

Reference	Designation	Trial start	Trial duration [weeks]	Trial participants, N	Women, N	Women [%]	Location	Approved/ experimental	Mean baseline MMSE	Mean baseline age [years]
Doody, 2013[32]	primary AD	2008	76	1534	820	53.46	WW	experimental	20.80	73.20
Vandenberghe, 2016[33]	primary AD	2008	78	1917	1155	60.25	WW	experimental	20.88	70.54
Gold, 2010[34]	primary AD	2007	24	553	348	62.93	WW	experimental	19.23	72.35
Coric, 2012[35]	primary AD	2009	24	209	100	47.85	WW	experimental	21.36	73.72
Salloway, 2014[36]	primary AD	2007	78	2204	1188	53.90	NoA	experimental	22.95	72.40
Egan, 2012[37]	primary AD	2006	4	144	79	54.86	NoA	experimental	22.15	74.05
Sevigny, 2008[38]	primary AD	2003	52	563	326	57.90	NoA	experimental	20.65	76.00
Cummings, 2012[39]	primary AD	2007	48	567	367	64.73	WW	approved	14.20	75.70
Burns, 2009[40]	primary AD	2003	26	407	329	80.84	EU	approved	8.95	83.60
Grossberg, 2013[41]	primary AD	2005	24	676	487	72.04	WW	approved	10.75	76.50
Green, 2009[42]	primary AD	2005	78	1649	840	50.94	NoA	experimental	23.30	74.60
Wischik, 2015[43]	primary AD	2004	24	321	172	53.58	WW	experimental	19.40	73.80
Xiao, 2017[44]	primary AD	2011	16	273	176	64.47	AS	experimental	17.43	72.19
Nakamura, 2011[45]	primary AD	2007	24	855	584	68.30	AS	approved	16.60	74.60
Gauthier, 2015[46]	primary AD	2009	12	203	104	51.23	NoA	experimental	17.30	76.60
Grove, 2014[47]	primary AD	2009	16	194	126	64.95	WW	experimental	19.95	71.80
Voss, 2018[48]	primary AD	2013	24	239	129	53.97	NoA	experimental	18.35	72.10
Rinne, 2017[49]	primary AD	2011	12	100	59	59.00	EU	experimental	18.50	72.00
Zhang, 2016[50]	primary AD	2011	24	501	279	55.69	AS	approved	16.30	70.10
Salloway, 2011[51]	primary AD	2007	78	351	197	56.13	NoA	experimental	20.42	73.35

© 2021 Martinkova J et al. *JAMA Network Open*.

Reference	Designation	Trial start	Trial duration [weeks]	Trial participants, N	Women, N	Women [%]	Location	Approved/ experimental	Mean baseline MMSE	Mean baseline age [years]
Galasko, 2014[52]	primary AD	2007	78	399	228	57.03	NoA	experimental	20.40	72.93
Rafii, 2011[53]	primary AD	2004	16	210	135	64.29	NoA	experimental	19.12	77.92
Maher-Edwards, 2015[54]	primary AD	2011	24	121	61	50.53	WW	experimental	22.85	73.00
Nakamura, 2015[55]	primary AD	2012	24	215	145	67.46	AS	approved	17.10	77.50
Vellas, 2011[56]	primary AD	2008	12	157	88	56.05	EU	experimental	19.21	76.30
Feldman, 2010[57]	primary AD	2002	72	614	319	51.97	NoA	experimental	21.85	73.59
Maher-Edwards, 2010[58]	primary AD	2005	24	357	207	57.96	WW	experimental	20.00	69.80
Cummings, 2018[59]	primary AD	2011	73	433	228	52.65	WW	experimental	21.73	70.70
Brody, 2016[60]	primary AD	2010	104	146	84	57.53	NoA	experimental	22.00	72.80
Harrington, 2011[61]	primary AD	2006	48	2822	1627	57.65	WW	experimental	18.30	73.66
Aisen, 2011[62]	primary AD	2004	78	1005	533	53.03	NoA	experimental	21.10	73.90
Homma, 2016[63]	primary AD	2012	24	340	236	69.41	AS	approved	8.70	76.00
Frölich, 2019[64]	primary prodromal	2015	12	452	231	51.11	WW	experimental	NA	73.60
Scheltens, 2018[65]	primary prodromal	2015	12	120	64	53.33	EU	experimental	25.00	71.40
Sevigny, 2016[66]	primary prodromal	2012	54	165	83	50.30	NoA	experimental	24.20	72.60
Ostrowiczki, 2017[67]	primary prodromal	2010	104	797	NA	NA	WW	experimental	25.70	70.36
Coric, 2015[68]	primary prodromal	2009	104	263	114	43.36	WW	experimental	27.00	71.70
Egan, 2019[69]	primary prodromal	2013	104	1454	686	47.18	WW	experimental	26.33	71.43
Schneeberger, 2015[70]	primary prodromal	2010	65	332	169	50.90	EU	experimental	23.30	70.40

© 2021 Martinkova J et al. *JAMA Network Open*.

Reference	Designation	Trial start	Trial duration [weeks]	Trial participants, N	Women, N	Women [%]	Location	Approved/ experimental	Mean baseline MMSE	Mean baseline age [years]
Craft, 2012[71]	primary prodromal	2006	17	106	45	42.46	NoA	experimental	NA	72.35
Ferris, 2011[72]	secondary	2007	24	1371	861	62.80	WW	approved	13.10	73.80
Doody, 2012[73]	secondary	2007	24	1434	901	62.83	WW	approved	13.11	73.80
Salloway, 2012[74]	secondary	2007	24	1434	901	62.83	WW	approved	13.11	73.80
Schmitt, 2013[75]	secondary	2007	24	1371	861	62.80	WW	approved	NA	73.80
Hager, 2016[76]	secondary	2008	104	2045	1325	64.79	EU	approved	19.00	73.00
Doody, 2015[77]	secondary	2008	76	1534	820	53.46	WW	experimental	NA	73.20
Liu, 2015[78]	secondary	2007	78	154	82	53.25	NoA	experimental	20.98	71.00
Samtani, 2015[79]	secondary	2007	78	154	82	53.25	NoA	experimental	NA	71.00
Molinuevo, 2015[80]	secondary	2007	48	568	368	64.79	WW	approved	NA	76.70
Grossberg, 2018[81]	secondary	2005	24	676	487	72.04	WW	approved	NA	76.50
Ohnishi, 2014[82]	secondary	2007	24	574	389	67.77	AS	approved	NA	75.20
Yoshida, 2020[83]	secondary	2011	73	NA	NA	NA	WW	experimental	NA	NA
Abushakra, 2017[84]	secondary	2004	78	257	144	56.03	WW	experimental	21.12	71.10

eTable 5: Overview of study details and extracted data. Tabular overview of main extracted variables for all included studies. Rounded to 0 decimal points, or to 2 decimal points (proportion of women in %, baseline age, baseline MMSE). Designation assigned as follows: primary AD = chronologically first publication publishing primary results of a trial for Alzheimer's disease dementia, primary prodromal = chronologically first publication publishing the main results of a trial for prodromal Alzheimer's disease, secondary = any subsequent publications from the same trial.

© 2021 Martinkova J et al. *JAMA Network Open*.

MMSE = mini mental state examination, WW = worldwide (2 continents or more), NoA = North America, AS = Asia, EU = Europe, NA = not available

eTable 6: Proportion of women in subgroups: non-parametric correlation matrix

	**MMSE**	**Year published**	**Year started**	**Age**	**Approved/experimental**	**Trial duration**	**Asia**	**Europe**	**North America**
Year published	rho=-0.02, p=0.89								
Year started	rho=-0.13, p=0.35	**rho=0.85, p=<0.001****							
Age	rho=-0.27, p=0.05	**rho=-0.31, p=0.02***	rho=-0.24, p=0.07						
Approved status	**rho=-0.56, p=<0.001****	rho=-0.18, p=0.19	rho=-0.09, p=0.52	**rho=0.32, p=0.02***					
Trial duration	**rho=0.35, p=0.008****	rho=0.09, p=0.52	rho=-0.03, p=0.84	**rho=-0.34, p=0.01***	rho=-0.06, p=0.68				
Asia	**rho=-0.39, p=0.003****	rho=0.00, p=0.99	rho=0.11, p=0.44	rho=-0.01, p=0.94	**rho=0.48, p=<0.001****	rho=-0.19, p=0.16			
Europe	rho=-0.15, p=0.28	rho=-0.07, p=0.60	rho=-0.07, p=0.63	rho=0.15, p=0.28	rho=0.16, p=0.23	rho=0.00, p=0.99			
North America	**rho=0.42, p=0.001****	rho=-0.06, p=0.64	rho=-0.21, p=0.12	rho=0.07, p=0.63	**rho=-0.28, p=0.04***	rho=0.17, p=0.20			
Proportion women	**rho=-0.62, p=<0.001****	rho=-0.23, p=0.09	rho=-0.12, p=0.37	rho=0.18, p=0.19	**rho=0.47, p=<0.001****	rho=-0.16, p=0.23	**rho=0.34, p=0.01***	**rho=0.28, p=0.04***	**rho=-0.49, p=<0.001****

eTable 6: Proportion of women in subgroups: non-parametric correlation matrix. Correlation matrix using non-parametric Spearman correlation. Significant in bold (* significance level 0.05, ** significance level 0.01). There are several statistically significant correlations, notably between the proportion of women and MMSE (rho=–0.62, p<0.001), approved/experimental status (rho=0.47, p<0.001), and all location variables (Asia: rho=0.34, p=0.01; Europe: rho=0.28, p=0.04; North America: rho=–0.49, p<0.001).

AD = Alzheimer's disease, MMSE = mini mental state examination

© 2021 Martinkova J et al. *JAMA Network Open*.

eTable 7: Multivariate mixed effect logistic regression model with probability of trial participant being a women as a dependent variable, Model 3 (location only): summary of fixed effects

	OR	Z value	p-value	CI
Intercept	1.47	9.14	**<0.001****	**(1.36,1.60)**
Location: Asia	1.28	2.34	**0.02***	**(1.04,1.57)**
Location: Europe	1.42	3.23	**0.001****	**(1.15,1.75)**
Location: North America	0.81	-2.92	**0.003****	**(0.71,0.93)**

eTable 7: Multivariate mixed effect logistic regression model with probability of trial participant being a woman as a dependent variable, Model 3 (location only): summary of fixed effects. Tabular representation of the multivariable mixed effect binomial regression of probability of trial participant being a woman on predictor variables, location variables included. Significant results in bold, significance level denoted by asterisks (* significance level 0.05, ** significance level 0.01).
All location predictor variables are statistically significant, with OR varying from to 0.81 for location in North America to 1.42 for location in Europe.
OR = odds ratio, CI = 95% confidence interval

eTable 8: Odds ratio of female enrolment by location: pairwise comparisons

	Estimate OR	Z value	p-value
Asia vs. worldwide	1.16	1.39	0.98
Europe vs. worldwide	1.26	2.45	0.08
North America vs. worldwide	0.81	-3.10	**0.01 ***
Europe vs. Asia	1.09	0.65	0.99
North America vs. Asia	0.70	-2.77	**0.03 ***
North America vs. Europe	0.65	-4.37	**<0.001 ****

eTable 8: Odds ratio of female enrolment by location: pairwise comparisons. eTable 8 shows results of pairwise comparisons the coefficients of different locations, using Bonferroni adjustment for multiple comparisons. The results show a solid correlation of North America with a lower probability of female enrollment (OR 0.81 vs. worldwide, 0.70 and 0.65 vs Asia and Europe respectively). Significant results in bold, significance level denoted by asterisks (* significance level 0.05, ** significance level 0.01).
OR = odds ratio

eTable 9: Reporting of sex-stratified data in primary studies (n=56)

	**N of studies, %**
Study protocol available	17 (30.4%)<br><br>Of these, sex-stratified analysis present in protocol in 8/17 (47.1%)
Sex-stratified analysis in Methods	8 (14.3%)
Sex-stratified data reported in Results	7 (12.5%)<br>Of these, sex differences observed in results in 1/7 (14.3%)

eTable 10: Bias assessment table

**Unique ID**	**Reference**	**Study ID**	**a**	**b**	**c**	**d**	**e**	**f**	**g**
1	Egan et al. 2018	6.1	LR	LR	LR	LR	LR	LR	LR
2	Schneider et al 2019	9	LR	LR	LR	LR	LR	LR	LR
3	Maher-Edwards 2015	12	LR	LR	LR	LR	LR	LR	LR
4	Lenz et al 2015	14	LR	LR	LR	LR	LR	NI	LR
5	Farlow et al 2010	15.1	LR	LR	LR	LR	LR	NI	LR
6	Doody et al 2008	17	LR	LR	LR	LR	LR	NI	LR
7	Pasquier et al 2016	20	LR	NI	LR	LR	LR	NI	LR
8	Lawlor 2018	24.1	LR	LR	LR	LR	LR	LR	LR
9	Gauthier 2016	31	LR	LR	LR	LR	LR	NI	LR
10	Frohlich 2019	34	LR	LR	LR	NI	LR	LR	LR
11	Scheltens 2018	40	LR	LR	LR	NI	LR	LR	LR
12	Hager 2014	42.1	LR	LR	LR	LR	HR	LR	LR
13	van Dyck 2019	46	LR	LR	LR	LR	LR	LR	LR
14	Honig 2018	47	LR	LR	LR	LR	LR	LR	LR
15	Atri 2018	51.1	LR	LR	LR	LR	LR	LR	LR
16	Maher-edwards 2010	52	LR	NI	LR	NI	LR	NI	HR
17	Gault 2016	55	LR	LR	LR	NI	LR	LR	LR
18	Florian 2016	56	LR	LR	LR	LR	LR	NI	LR
19	Nave 2017	59	LR	LR	LR	LR	LR	LR	LR
20	Sevigny 2016	60.1	LR	LR	HR	HR	LR	NI	LR
21	Fullerton 2018	62	LR	LR	LR	LR	LR	LR	LR
22	Doody 2014	67.1	LR	LR	LR	LR	LR	LR	LR
23	Marek 2014	69	LR	LR	LR	LR	HR	NI	LR
24	Ostrowitzki 2017	71.2	LR	NI	LR	NI	LR	NI	LR

© 2021 Martinkova J et al. *JAMA Network Open*.

Unique ID	Reference	Study ID	a	b	c	d	e	f	g
25	Wilckinson 2014	75	LR	LR	LR	LR	LR	NI	LR
26	Relkin 2017	76	LR	LR	LR	LR	LR	NI	LR
27	Gault 2015	78	LR	NI	LR	LR	LR	NI	LR
28	Alvarez 2011	82	LR	LR	LR	LR	LR	NI	LR
29	Doody 2013	84.1	LR	LR	LR	HR	LR	LR	LR
30	Coric 2015	89	LR	LR	LR	LR	NI	LR	LR
31	Vanderberghe 2016	90.1	LR	LR	LR	LR	HR	NI	LR
32	Gold 2010	92	LR	NI	LR	LR	LR	NI	LR
33	Coric 2012	93	LR	LR	LR	LR	NI	NI	LR
34	Salloway 2014	94.1	LR	LR	HR	LR	LR	LR	LR
35	Egan 2012	99	LR	NI	LR	LR	LR	NI	LR
36	Sevigny 2008	108.1	LR	LR	LR	LR	NI	NI	LR
37	Cummings 2012	112.1	LR	NI	LR	LR	LR	NI	LR
38	Burns 2009	125	LR	LR	LR	LR	LR	NI	LR
39	Egan 2019	131.2	LR	LR	LR	LR	HR	LR	LR
40	Grossberg 2013	134.1	LR	LR	LR	LR	LR	NI	LR
41	Green 2009	161	LR	LR	LR	NI	LR	NI	LR
42	Wischik 2015	174	LR	LR	HR	LR	LR	LR	LR
43	Xiao 2017	199	LR	NI	LR	LR	LR	NI	LR
44	Nakamura et al 2011	200	LR	NI	LR	LR	LR	NI	LR
45	Gauthier 2015	208	LR	NI	LR	LR	LR	NI	LR
46	Grove 2014	210	LR	LR	LR	LR	LR	NI	LR
47	Voss 2018	211	LR	LR	LR	LR	HR	LR	LR
48	Rinne 2017	212	LR	LR	LR	LR	LR	NI	LR
49	Zin Chang 2015	214	LR	LR	HR	LR	LR	NI	LR
50	Salloway 2011	220	LR	LR	LR	LR	HR	NI	HR
51	Galasko 2014	223	LR	NI	LR	LR	LR	NI	LR
52	Schneeberger 2015	226	LR	NI	LR	NI	LR	NI	LR
53	Rafii 2011	231	LR	NI	LR	LR	LR	NI	LR
54	Maher Edwards 2015	8	LR	NI	LR	LR	LR	NI	LR
56	Craft 2012	175	LR	NI	LR	LR	LR	NI	HR
58	Nakamura 2015	230	LR	LR	LR	LR	LR	NI	LR
59	Vellas 2011	215	LR	NI	LR	LR	LR	NI	HR
60	Feldman et al. 2010	275	LR	LR	LR	LR	LR	NI	LR
61	Maher-Edwards et al. 2010	276	LR	LR	LR	LR	LR	NI	LR
62	Cummings et al. 2018	278	LR	LR	LR	LR	LR	NI	LR
63	Brody et al. 2016	279	LR	LR	LR	LR	LR	NI	LR

© 2021 Martinkova J et al. *JAMA Network Open*.

Unique ID	Reference	Study ID	a	b	c	d	e	f	g
64	Harrington et al. 2011	281	LR	NI	LR	LR	LR	NI	LR
65	Aisen et al. 2011	295.1	LR	LR	LR	LR	HR	NI	LR
67	Homma et al. 2016	309	LR	LR	LR	LR	LR	NI	LR

eTable 10: Bias assessment table. Tabular representation of risk of bias in individual primary studies (56 publications on AD and 8 on prodromal AD) using a modified version of the Cochrane risk of bias tool RoB2. a: random sequence generation (selection bias); b: allocation concealment (selection bias); c: blinding of participants and personnel (performance bias); d: blinding of outcome assessment (detection bias); e: incomplete outcome data (attrition bias); f: selective reporting (reporting bias); g: other sources of bias. Each study was rated as having low (LR), high (HR), or unclear because of not enough information (NI) risk of bias

eReferences

1. Gendered Innovations in Science, Health & Medicine, Engineering, and E. Terminology. *Stanford University* (2011). Available at: https://genderedinnovations.stanford.edu/terms.html.
2. R Core Team. R: A Language and Environment for Statistical Computing. (2020).
3. Pasek, J. & Tahk, A. weights: Weighting and Weighted Statistics. *R package version 1.0.1* (2020).
4. Dorai-Raj, S. binom: Binomial Confidence Intervals For Several Parameterizations. *R package version 1.1-1* (2014).
5. Wickham, H. ggplot2: Elegant Graphics for Data Analysis. *Springer-Verlag New York* (2016).
6. Gordon, M. & Lumley, T. forestplot: Advanced Forest Plot Using 'grid' Graphics. *R package version 1.10.1* (2020).
7. Yoshida, K. tableone: Create 'Table 1' to Describe Baseline Characteristics. *R package version 0.10.0* (2019).
8. Egan, M. F. *et al.* Randomized trial of Verubecestat for mild-to-moderate Alzheimer's disease. *N. Engl. J. Med.* **378**, 1691–1703 (2018).
9. Schneider, L. S. *et al.* Safety and Efficacy of Edonerpic Maleate for Patients with Mild to Moderate Alzheimer Disease: A Phase 2 Randomized Clinical Trial. *JAMA Neurol.* **76**, 1330–1339 (2019).
10. Maher-Edwards, G. *et al.* Two randomized controlled trials of SB742457 in mild-to-moderate Alzheimer's disease. *Alzheimer's Dement. Transl. Res. Clin. Interv.* **1**, 23–36 (2015).
11. Lenz, R. A. *et al.* Adaptive, Dose-finding Phase 2 Trial Evaluating the Safety and Efficacy of ABT-089 in Mild to Moderate Alzheimer Disease. *Alzheimer Dis. Assoc. Disord.* **29**, 192–199 (2015).
12. Farlow, M. R. *et al.* Effectiveness and tolerability of high-dose (23 mg/d) versus standard-dose (10 mg/d) donepezil in moderate to severe Alzheimer's disease: A 24-week, randomized, double-blind study. *Clin. Ther.* **32**, 1234–1251 (2010).
13. Doody, R. S. *et al.* Effect of dimebon on cognition, activities of daily living, behaviour,

© 2021 Martinkova J et al. *JAMA Network Open*.

and global function in patients with mild-to-moderate Alzheimer's disease: a randomised, double-blind, placebo-controlled study. *Lancet* **372**, 207–215 (2008).
14. Pasquier, F. *et al.* Two phase 2 multiple ascending-dose studies of vanutide cridificar (ACC-001) and QS-21 adjuvant in mild-to-moderate Alzheimer's disease. *J. Alzheimer's Dis.* **51**, 1131–1143 (2016).
15. Lawlor, B. *et al.* Nilvadipine in mild to moderate Alzheimer disease: A randomised controlled trial. *PLoS Med.* **15**, 1–20 (2018).
16. Gauthier, S. *et al.* Efficacy and safety of tau-aggregation inhibitor therapy in patients with mild or moderate Alzheimer's disease: a randomised, controlled, double-blind, parallel-arm, phase 3 trial. *Lancet (London, England)* **388**, 2873–2884 (2016).
17. Hager, K. *et al.* Effects of galantamine in a 2-year, randomized, placebo-controlled study in Alzheimer's disease. *Neuropsychiatr. Dis. Treat.* **10**, 391–401 (2014).
18. Van Dyck, C. H. *et al.* Effect of AZD0530 on Cerebral Metabolic Decline in Alzheimer Disease: A Randomized Clinical Trial. *JAMA Neurol.* **76**, 1219–1229 (2019).
19. Honig, L. S. *et al.* Trial of Solanezumab for mild dementia due to Alzheimer's disease. *N. Engl. J. Med.* **378**, 321–330 (2018).
20. Atri, A. *et al.* Effect of idalopirdine as adjunct to cholinesterase inhibitors on change in cognition in patients with Alzheimer disease three randomized clinical trials. *JAMA - J. Am. Med. Assoc.* **319**, 130–142 (2018).
21. Maher-Edwards, G. *et al.* SB-742457 and donepezil in Alzheimer disease: A randomized, placebo-controlled study. *Int. J. Geriatr. Psychiatry* **26**, 536–544 (2011).
22. Gault, L. M. *et al.* ABT-126 monotherapy in mild-to-moderate Alzheimer's dementia: Randomized double-blind, placebo and active controlled adaptive trial and open-label extension. *Alzheimer's Res. Ther.* **8**, 1–13 (2016).
23. Florian, H. *et al.* Efficacy and safety of ABT-126 in subjects with mild-to-moderate Alzheimer's disease on stable doses of acetylcholinesterase inhibitors: A randomized, double-blind, placebo-controlled study. *J. Alzheimer's Dis.* **51**, 1237–1247 (2016).
24. Nave, S. *et al.* Sembragiline in Moderate Alzheimer's Disease: Results of a Randomized, Double-Blind, Placebo-Controlled Phase II Trial (MAyflOwer RoAD). *J. Alzheimer's Dis.* **58**, 1217–1228 (2017).
25. Fullerton, T. *et al.* A Phase 2 clinical trial of PF-05212377 (SAM-760) in subjects with mild to moderate Alzheimer's disease with existing neuropsychiatric symptoms on a stable daily dose of donepezil. *Alzheimer's Res. Ther.* **10**, 1–10 (2018).
26. Doody, R. S. *et al.* Phase 3 trials of solanezumab for mild-to-moderate Alzheimer's disease. *N. Engl. J. Med.* **370**, 311–321 (2014).
27. Marek, G. J. *et al.* Efficacy and safety evaluation of HSD-1 inhibitor ABT-384 in Alzheimer's disease. *Alzheimer's Dement.* **10**, S364–S373 (2014).
28. Wilkinson, D., Windfeld, K. & Colding-Jørgensen, E. Safety and efficacy of idalopirdine, a 5-HT6 receptor antagonist, in patients with moderate Alzheimer's disease (LADDER): A randomised, double-blind, placebo-controlled phase 2 trial. *Lancet Neurol.* **13**, 1092–1099 (2014).
29. Relkin, N. R. *et al.* A phase 3 trial of IV immunoglobulin for Alzheimer disease. *Neurology* **88**, 1768–1775 (2017).
30. Gault, L. M. *et al.* A phase 2 randomized, controlled trial of the α7 agonist ABT-126 in mild-to-moderate Alzheimer's dementia. *Alzheimer's Dement. Transl. Res. Clin. Interv.* **1**, 81–90 (2015).
31. A. Alvarez, X. *et al.* Combination Treatment in Alzheimers Disease: Results of a

© 2021 Martinkova J et al. *JAMA Network Open*.

Randomized, Controlled Trial with Cerebrolysin and Donepezil. *Curr. Alzheimer Res.* **8**, 583–591 (2011).
32. Doody, R. S. *et al.* A phase 3 trial of semagacestat for treatment of Alzheimer's disease. *N. Engl. J. Med.* **369**, 341–350 (2013).
33. Vandenberghe, R. *et al.* Bapineuzumab for mild to moderate Alzheimer's disease in two global, randomized, phase 3 trials. *Alzheimers. Res. Ther.* **8**, 18 (2016).
34. Gold, M. *et al.* Rosiglitazone monotherapy in mild-to-moderate Alzheimer's disease: results from a randomized, double-blind, placebo-controlled phase III study. *Dement. Geriatr. Cogn. Disord.* **30**, 131–146 (2010).
35. Coric, V. *et al.* Safety and tolerability of the γ-secretase inhibitor avagacestat in a phase 2 study of mild to moderate Alzheimer disease. *Arch. Neurol.* **69**, 1430–1440 (2012).
36. Salloway, S. *et al.* Two phase 3 trials of bapineuzumab in mild-to-moderate Alzheimer's disease. *N. Engl. J. Med.* **370**, 322–333 (2014).
37. Michael Egan *et al.* Pilot Randomized Controlled Study of a Histamine Receptor Inverse Agonist in the Symptomatic Treatment of AD. *Curr. Alzheimer Res.* **9**, 481–490 (2012).
38. Sevigny, J. J. *et al.* Growth hormone secretagogue MK-677: No clinical effect on AD progression in a randomized trial. *Neurology* **71**, 1702–1708 (2008).
39. Cummings, J. *et al.* Randomized, double-blind, parallel-group, 48-week study for efficacy and safety of a higher-dose rivastigmine patch (15 vs. 10 cm) in Alzheimer's disease. *Dement. Geriatr. Cogn. Disord.* **33**, 341–353 (2012).
40. Burns, A. *et al.* Safety and efficacy of galantamine (Reminyl) in severe Alzheimer's disease (the SERAD study): a randomised, placebo-controlled, double-blind trial. *Lancet Neurol.* **8**, 39–47 (2009).
41. Grossberg, G. T. *et al.* The safety, tolerability, and efficacy of once-daily memantine (28 mg): A multinational, randomized, double-blind, placebo-controlled trial in patients with moderate-to-severe alzheimer's disease taking cholinesterase inhibitors. *CNS Drugs* **27**, 469–478 (2013).
42. Green, R. C. *et al.* Effect of tarenflurbil on cognitive decline and activities of daily living in patients with mild Alzheimer disease: a randomized controlled trial. *JAMA* **302**, 2557–2564 (2009).
43. Wischik, C. M. *et al.* Tau aggregation inhibitor therapy: An exploratory phase 2 study in mild or moderate Alzheimer's disease. *J. Alzheimer's Dis.* **44**, 705–720 (2015).
44. Xiao, S. *et al.* Efficacy and safety of a novel acetylcholinesterase inhibitor octohydroaminoacridine in mild-tomoderate Alzheimer's disease: A Phase II multicenter randomised controlled trial. *Age Ageing* **46**, 767–773 (2017).
45. Nakamura, Y. *et al.* A 24-Week, Randomized, Double-Blind, Placebo-Controlled Study to Evaluate the Efficacy, Safety and Tolerability of the Rivastigmine Patch in Japanese Patients with Alzheimer's Disease. *Dement. Geriatr. Cogn. Dis. Extra* **1**, 163–179 (2011).
46. Gauthier, S. *et al.* Effects of the Acetylcholine Release Agent ST101 with Donepezil in Alzheimer's Disease: A Randomized Phase 2 Study. *J. Alzheimer's Dis.* **48**, 473–481 (2015).
47. Grove, R. *et al.* A Randomized, Double-Blind, Placebo-Controlled, 16-Week Study of the H_3 Receptor Antagonist, GSK239512 as a Monotherapy in Subjects with Mild-to-Moderate Alzheimer's Disease. *Curr. Alzheimer Res.* **11**, 47–58 (2014).

© 2021 Martinkova J et al. *JAMA Network Open*.

48. Voss, T. *et al.* Randomized, controlled, proof-of-concept trial of MK-7622 in Alzheimer's disease. *Alzheimer's Dement. Transl. Res. Clin. Interv.* **4**, 173–181 (2018).
49. Rinne, J. O. *et al.* Tolerability of ORM-12741 and effects on episodic memory in patients with Alzheimer's disease. *Alzheimer's Dement. Transl. Res. Clin. Interv.* **3**, 1–9 (2017).
50. Zhang, Z. X. *et al.* Rivastigmine Patch in Chinese Patients with Probable Alzheimer's disease: A 24-week, Randomized, Double-Blind Parallel-Group Study Comparing Rivastigmine Patch (9.5 mg/24 h) with Capsule (6 mg Twice Daily). *CNS Neurosci. Ther.* **22**, 488–496 (2016).
51. Salloway, S. *et al.* A phase 2 randomized trial of ELND005, scyllo-inositol, in mild to moderate Alzheimer disease Supplemental data at www.neurology.org. *Neurol.* ® **77**, 1253–1262 (2011).
52. Galasko, D. *et al.* Clinical trial of an inhibitor of RAGE-Ab interactions in Alzheimer disease. *Neurology* **82**, 1536–1542 (2014).
53. Rafii, M. S. *et al.* A phase II trial of huperzine A in mild to moderate Alzheimer disease. *Neurology* **76**, 1389–1394 (2011).
54. Maher-Edwards, G., De'Ath, J., Barnett, C., Lavrov, A. & Lockhart, A. A 24-week study to evaluate the effect of rilapladib on cognition and cerebrospinal fluid biomarkers of Alzheimer's disease. *Alzheimer's Dement. Transl. Res. Clin. Interv.* **1**, 131–140 (2015).
55. Nakamura, Y. *et al.* A 24-Week, Randomized, Controlled Study to Evaluate the Tolerability, Safety and Efficacy of 2 Different Titration Schemes of the Rivastigmine Patch in Japanese Patients with Mild to Moderate Alzheimer's Disease. *Dement. Geriatr. Cogn. Dis. Extra* **5**, 361–374 (2015).
56. Vellas, B. *et al.* EHT0202 in Alzheimer's Disease: a 3-Month, Randomized, Placebo-Controlled, Double-Blind Study. *Curr. Alzheimer Res.* **999**, 1–10 (2011).
57. Feldman, H. H. *et al.* Randomized controlled trial of atorvastatin in mild to moderate Alzheimer disease: LEADe. *Neurology* **74**, 956–964 (2010).
58. Maher-Edwards, G. *et al.* Double-Blind, Controlled Phase II Study of a 5-HT6 Receptor Antagonist, SB-742457, in Alzheimer's Disease. *Curr. Alzheimer Res.* **999**, 1–12 (2010).
59. Cummings, J. L. *et al.* A phase 2 randomized trial of crenezumab in mild to moderate Alzheimer disease. *Neurology* **90**, E1889–E1897 (2018).
60. Brody, M. *et al.* A Phase II, Randomized, Double-Blind, Placebo-Controlled Study of Safety, Pharmacokinetics, and Biomarker Results of Subcutaneous Bapineuzumab in Patients with mild to moderate Alzheimer's disease. *J. Alzheimer's Dis.* **54**, 1509–1519 (2016).
61. Harrington, C. *et al.* Rosiglitazone does not improve cognition or global function when used as adjunctive therapy to AChE inhibitors in mild-to-moderate Alzheimer's disease: two phase 3 studies. *Curr. Alzheimer Res.* **8**, 592–606 (2011).
62. Aisen, P. S. *et al.* Tramiprosate in mild-to-moderate Alzheimer's disease - A randomized, double-blind, placebo-controlled, multi-centre study (the alphase study). *Arch. Med. Sci.* **7**, 102–111 (2011).
63. Homma, A., Atarashi, H., Kubota, N., Nakai, K. & Takase, T. Efficacy and Safety of Sustained Release Donepezil High Dose versus Immediate Release Donepezil Standard Dose in Japanese Patients with Severe Alzheimer's Disease: A Randomized, Double-Blind Trial. *J. Alzheimer's Dis.* **52**, 345–357 (2016).
64. Frölich, L. *et al.* Evaluation of the efficacy, safety and tolerability of orally administered BI 409306, a novel phosphodiesterase type 9 inhibitor, in two

© 2021 Martinkova J et al. *JAMA Network Open*.

randomised controlled phase II studies in patients with prodromal and mild Alzheimer's disease. *Alzheimer's Res. Ther.* **11**, 1–11 (2019).
65. Scheltens, P. *et al.* Safety, tolerability and efficacy of the glutaminyl cyclase inhibitor PQ912 in Alzheimer's disease: Results of a randomized, double-blind, placebo-controlled phase 2a study. *Alzheimer's Res. Ther.* **10**, 1–14 (2018).
66. Sevigny, J. *et al.* The antibody aducanumab reduces Aβ plaques in Alzheimer's disease. *Nature* **537**, 50–56 (2016).
67. Ostrowitzki, S. *et al.* A phase III randomized trial of gantenerumab in prodromal Alzheimer's disease. *Alzheimers. Res. Ther.* **9**, 95 (2017).
68. Coric, V. *et al.* Targeting prodromal Alzheimer disease with avagacestat: A randomized clinical trial. *JAMA Neurol.* **72**, 1324–1333 (2015).
69. Egan, M. F. *et al.* Randomized trial of verubecestat for prodromal Alzheimer's disease. *N. Engl. J. Med.* **380**, 1408–1420 (2019).
70. Schneeberger, A., Hendrix, S., Ellison, N., Bürger, V. & Dubois, B. Additional results from a phase ii study to assess the clinical and immunological activity, safety, and tolerability of affitope® ad02 in patients with early Alzheimer's disease (AD). *Alzheimer's Dement.* **11**, P276 (2015).
71. Craft, S. Intranasal Insulin Therapy for Alzheimer Disease and Amnestic Mild Cognitive Impairment. *Arch. Neurol.* **69**, 29 (2012).
72. Ferris, S. H. *et al.* Analyzing the impact of 23 mg/day donepezil on language dysfunction in moderate to severe Alzheimer's disease. *Alzheimer's Res. Ther.* **3**, 22 (2011).
73. Doody, R. S. *et al.* Efficacy and safety of donepezil 23 mg versus donepezil 10 mg for moderate-to-severe Alzheimer's disease: A subgroup analysis in patients already taking or not taking concomitant memantine. *Dement. Geriatr. Cogn. Disord.* **33**, 164–173 (2012).
74. Salloway, S. *et al.* Subgroup analysis of US and non-US patients in a global study of high-dose donepezil (23 mg) in moderate and severe Alzheimer's disease. *Am. J. Alzheimers. Dis. Other Demen.* **27**, 421–432 (2012).
75. Schmitt, F. A., Saxton, J., Ferris, S. H., Mackell, J. & Sun, Y. Evaluation of an 8-item Severe Impairment Battery (SIB-8) vs. the full SIB in moderate to severe Alzheimer's disease patients participating in a donepezil study. *Int. J. Clin. Pract.* **67**, 1050–1056 (2013).
76. Hager, K. *et al.* Effect of concomitant use of memantine on mortality and efficacy outcomes of galantamine-treated patients with Alzheimer's disease: post-hoc analysis of a randomized placebo-controlled study. *Alzheimer's Res. Ther.* **8**, 1–10 (2016).
77. Doody, R. S. *et al.* Peripheral and central effects of γ-secretase inhibition by semagacestat in Alzheimer's disease. *Alzheimer's Res. Ther.* **7**, 1–7 (2015).
78. Liu, E. *et al.* Amyloid-β 11C-PiB-PET imaging results from 2 randomized bapineuzumab phase 3 AD trials. *Neurology* **85**, 692–700 (2015).
79. Samtani, M. N. *et al.* Alzheimer's disease assessment scale-cognitive 11-item progression model in mild-to-moderate Alzheimer's disease trials of bapineuzumab. *Alzheimer's Dement. Transl. Res. Clin. Interv.* **1**, 157–169 (2015).
80. Molinuevo, J. L. *et al.* Responder analysis of a randomized comparison of the 13.3 mg/24 h and 9.5 mg/24 h rivastigmine patch. *Alzheimer's Res. Ther.* **7**, 1–6 (2015).
81. Grossberg, G. T. *et al.* Memantine ER Maintains Patient Response in Moderate to Severe Alzheimer's Disease: Post Hoc Analyses From a Randomized, Controlled,

© 2021 Martinkova J et al. *JAMA Network Open*.

Clinical Trial of Patients Treated With Cholinesterase Inhibitors. *Alzheimer Dis. Assoc. Disord.* **32**, 173–178 (2018).
82. Ohnishi, T. *et al.* The prediction of response to Galantamine treatment in Patients with mild to moderate Alzheimer's Disease. *Curr. Alzheimer Res.* **11**, 110–118 (2014).
83. Yoshida, K. *et al.* Pharmacokinetics and pharmacodynamic effect of crenezumab on plasma and cerebrospinal fluid beta-amyloid in patients with mild-to-moderate Alzheimer's disease. *Alzheimer's Res. Ther.* **12**, 1–12 (2020).
84. Abushakra, S. *et al.* Clinical Effects of Tramiprosate in APOE4/4 Homozygous Patients with Mild Alzheimer's Disease Suggest Disease Modification Potential. *J. Prev. Alzheimer's Dis.* **4**, 149–156 (2017).

© 2021 Martinkova J et al. *JAMA Network Open*.
